# miRNA expression atlas in male rat

**DOI:** 10.1038/sdata.2014.5

**Published:** 2014-05-27

**Authors:** Keiichi Minami, Takeki Uehara, Yuji Morikawa, Ko Omura, Masayuki Kanki, Akira Horinouchi, Atsushi Ono, Hiroshi Yamada, Yasuo Ohno, Tetsuro Urushidani

**Affiliations:** 1 Exploratory Research Laboratories, Tsukuba Research Institute, Ono Pharmaceutical Co., Ltd., 17-2 Wadai, Tsukuba-shi, Ibaraki 300-4247, Japan; 2 Drug Developmental Research Laboratories, Shionogi & Co., Ltd., 3-1-1 Futaba-cho, Toyonaka, Osaka 561-0825, Japan; 3 Toxicogenomics Informatics Project, National Institute of Biomedical Innovation, 7-6-8 Asagi, Ibaraki, Osaka 567-0085, Japan; 4 Drug Safety Research Laboratories, Astellas Pharma Inc., 2-1-6, Kashima, Yodogawa-ku, Osaka 532-8514, Japan; 5 Chemistry, Manufacturing and Controls Center, Takeda Pharmaceutical Company Limited, 17-85, Jusohonmachi 2-chome, Yodogawaku, Osaka 532-8686, Japan; 6 National Institute of Health Sciences, 1-18-1 Kamiyoga, Setagaya-ku, Tokyo 158-8501, Japan; 7 Department of Pathophysiology, Faculty of Pharmaceutical Sciences, Doshisha Women’s College of Liberal Arts, Kodo, Kyotanabe, Kyoto 610-0395, Japan

## Abstract

MicroRNAs (miRNAs) are small (~22 nucleotide) noncoding RNAs that play pivotal roles in regulation of gene expression. The value of miRNAs as circulating biomarkers is now broadly recognized; such tissue-specific biomarkers can be used to monitor tissue injury and several pathophysiological conditions in organs. In addition, miRNA profiles of normal organs and tissues are important for obtaining a better understanding of the source of modulated miRNAs in blood and how those modulations reflect various physiological and toxicological conditions. This work was aimed at creating an miRNA atlas in rats, as part of a collaborative effort with the Toxicogenomics Informatics Project in Japan (TGP2). We analyzed genome-wide miRNA profiles of 55 different organs and tissues obtained from normal male rats using miRNA arrays. The work presented herein represents a comprehensive dataset derived from normal samples profiled in a single study. Here we present the whole dataset with miRNA profiles of multiple organs, as well as precise information on experimental procedures and organ-specific miRNAs identified in this dataset.

## Background & Summary

MicroRNAs (miRNAs) are small (~ 22 nucleotide) noncoding RNAs that play pivotal roles in regulation of gene expression; miRNAs bind via complementary base-pairing to target transcripts to repress translation or promote mRNA degradation. Major miRNAs are highly conserved across species, allowing translation of biomarkers to the clinic. The machinery for miRNA-mediated gene regulation is conserved from plants to humans, and miRNAs are encoded by their respective genes. As with other regulatory molecules, miRNAs are frequently subject to changes in expression level due to a large range of physiological processes, such as development, immune responses, metabolism and diseases, as well as toxicological outcomes^1^. Mounting evidence indicates that miRNAs are frequently overexpressed or downregulated as a result of cancer, obesity, diabetes, inflammation, neurological disorders, cardiovascular diseases or autoimmune diseases^2–7,2–7,2–7,2–7,2–7,2–7^. Previous studies have shown that distinct miRNA signatures can be assigned to particular organs and tumor types^8^. For example, miR-21 is ubiquitously expressed and upregulated in various types of cancer, including lymphoma, lung, prostate and colorectal cancers among others^9^. Furthermore, tumor cells have been shown to release tumor-specific miRNAs into systemic circulation. Because of the fundamental roles played by miRNAs in cellular functions, the potential for miRNAs as novel therapeutic targets is now widely recognized. Several candidate therapeutic miRNAs have progressed into clinical and preclinical development; for example, antisense miR-122 is being developed as a treatment for hepatitis C virus, miR-208/499 for chronic heart failure, miR-195 for myocardial infarction and miR-34 and let-7 for cancer^10,11^. However, serious obstacles obstructing the development of miRNA-based therapies remain, including lack of tissue specificity and risks of systemic toxicity. Characterizing miRNA expression profiles of normal organs and tissues may improve our understanding of the etiology of diseased organs and of organ- and tissue-specificity of miRNAs, and the success rate of development of new miRNA-based therapeutics.

Additionally, miRNAs have the potential to be useful biomarkers for monitoring physiological conditions and tissue injury. Upon tissue injury, miRNAs are released into systemic circulation or other body fluids; upon release, these miRNAs can be detected in small-volume samples via specific and sensitive quantitative real-time PCR. The precise mechanisms underlying the remarkable stability of miRNAs in the RNase-rich environment of blood are not well understood; nevertheless, miRNAs persist and are remarkably stable in blood; and these miRNAs can persist in an encapsulated state in exosomes or in protein complexes with carriers such as Argonaute 2 and Nucleophosmin or the High Density Lipoprotein^12^. Moreover, several studies have successfully identified circulating miRNA-based biomarkers; for example, Mitchell *et al.*^13^ found that miR-141 was highly elevated in serum from patients with prostate cancer and hypothesized that this miRNA may be useful as a diagnostic marker. Similarly, Wang *et al.*^14^ found that miR-122a, which is specifically expressed in the liver, was circulating systemically in mice with acetaminophen- (APAP-) induced hepatotoxicity; the authors reported that miR-122a and miR-192 were detected in plasma as early as the point when elevated alanine aminotransferase activity was found evident. Laterza *et al.*^15^ also reported that plasma miRNA measurements could be useful for monitoring tissue injury in the liver, muscle and brain. Even in clinical research, plasma miR-122a was significantly elevated in APAP-induced hepatitis patients^16^. These reports clearly indicate that circulating organ-specific miRNAs can serve as useful biomarkers for tissue injury and disease status in various organs. The organ-specificity of circulating miRNAs, or in other words, the detailed tissue distribution of miRNAs, is important information to be used in obtaining biomarkers for organ toxicity, although desirable properties of biomarkers vary with intended use. In addition, miRNA profiles of normal organs and tissues are important for gaining a better understanding of the source of modulated miRNAs in blood and in gaining an understanding of how those modulations reflect various physiological and toxicological conditions.

To date, organ-specific miRNA profiles have been reported for several animal species, including human, mouse and rat^17–20,17–20,17–20,17–20^. However, the amount of data currently available is still inadequate, since there are only limited datasets with profiles in a limited number of organs. The main objectives of our data analysis were (i) to establish the validity of this dataset and (ii) to demonstrate organ-specific miRNAs identified in this dataset. In this report, we present a large-scale reference dataset constituting genome-wide miRNA profiles for 55 normal rat organs or tissues. The validity of this dataset was confirmed by comprehensive statistical analysis. We ultimately identified several organ-specific miRNAs in rats. These organ-specific miRNAs can potentially be used as biomarkers for identifying the origin of metastatic tumors and for monitoring toxicity in targeted organs. Furthermore, establishing combinations of organ-specific miRNA measurements may be a novel biomarker for monitoring simultaneous impairment in multiple organs. Our data also support the hypothesis that specificity of miRNA expression is conserved among different species, since the majority of organ-specific miRNAs identified in this rat study were also confirmed as organ-specific in humans. The value of miRNAs as novel therapeutic targets and circulating biomarkers to monitor tissue injury and several pathophysiological conditions in organs is now broadly recognized. In the course of development of miRNAs for practical use as targeted therapeutics and biomarkers, there is no doubt about the importance of open access large-scale datasets for a free miRNA atlas for normal organs/tissues and comparative data analysis. The work presented herein represents a comprehensive dataset derived from normal samples profiled in a single study. We believe that our dataset will be of particular value to both basic and translational scientists in biological and biomedical sciences, especially for novel target discovery and biomarker identification.

## Methods

### Animal experiments

For each miRNA microarray experiment, 9-week-old male Sprague-Dawley rats were obtained from Charles River Japan, Inc. (Kanagawa, Japan). After a 7-day quarantine and acclimatization period, 10-week-old animals were used (*N*=6). The animals were individually housed in stainless-steel cages in an animal room set to the following conditions: 12 h (7:00–19:00) light phase; ventilation rate, 12/h; temperature, 20 °C–26 °C; and relative humidity, 35–75%. Each animal had free access to water and pellet diet (CRF-1, sterilized by radiation, Oriental Yeast Co., Ltd., Tokyo, Japan). Each animal was anesthetized with ether. The animals were divided into 2 groups to compare the effects of peripheral blood cells in organs; 3 animals were perfused with saline to remove blood from all organs, while the remaining 3 animals that were not perfused were subjected to collection of only the heart (atrium and interventricular septum), kidney, liver and lung. All organs evaluated in this study are listed in Table 1 (available online only). Experimental protocols were reviewed and approved by the Ethics Review Committee for Animal Experimentation of the National Institute of Health Sciences.

### RNA extraction and miRNA microarray analysis

RNA was prepared using the miRNeasy kit (QIAGEN, Hilden, Germany), according to the manufacturer’s instructions. RNA was quantified using a DU-7400 spectrophotometer (Beckman Coulter, Brea, CA) and quality was monitored with the Agilent 2100 Bioanalyzer (Agilent Technologies, Palo Alto, CA). Cyanine-3 (Cy-3) labeled RNA was prepared from 0.1 μg RNA using the miRNA Complete Labeling and Hyb Kit (Agilent Technologies), according to the manufacturer’s instructions. The entire volume of Cy3-labeled RNA was incubated at 100 °C for 5 min in a 45 μl reaction volume containing 1x Agilent blocking agent, Hybridization Spike-In and Hi-RPM Hybridization Buffer. Upon completion of this incubation, samples were hybridized to an 8×15 k customized Agilent Rat miRNA microarray containing both miRBase 15.0 and 16.0 probes for 20 h at 55 °C on a rotating rack in an Agilent hybridization oven. After hybridization, microarrays were washed for 5 min at room temperature with GE Wash Buffer 1 (Agilent Technologies) and then for 5 min at 37 °C with GE Wash buffer 2 (Agilent Technologies); slides were then dried immediately. Immediately after this washing and drying step, slides were scanned using an Agilent DNA Microarray Scanner (G2565AA) at the following settings: one-color scan for 8×16 k array slides, scan area, 61×21.6 mm; scan resolution, 5 μm; dye channel, green; and extended dynamic range scan mode (Hi=100%, Lo=5%).

### Microarray data analysis

Expressionist analysis software Ver. 7.6 (Genedata AG, Basel, Switzerland) was used for normalization, principal component analysis (PCA) and hierarchical clustering. First, all signal intensities were scaled to the 75th percentile of the median of the dynamic target value for each array using the central tendency normalization method. Next, the following two statistical parameters were calculated for each probe: (i) *P*-value of the Shapiro-Wilk test with Bonferroni correction (R software) and (ii) maximal signal intensity. Probes were then filtered by a *P*-value of <0.05 and a maximal signal intensity of >100. To identify organ-specific miRNAs, expression profiles of filtered probes were further analyzed by model-based clustering using the ‘mclust’ R package (http://cran.r-project.org/web/packages/mclust/index.html). For this analysis, a histogram was created to determine the distribution of expression values among different organs for each gene. For multiple distributions, the presence of an isolated peak was considered attributable to organ-specific expression of miRNAs. Genes with isolated peaks consisting of less than 37 microarrays (20% of all microarrays) in the histogram were selected as candidate miRNAs that were specifically expressed in one or a few organs.

## Data Records

Microarray data are available in the NCBI Gene Expression Omnibus (GEO), accession GSE52754 (Data Citation 1). This accession contains matrix files of normalized miRNA expression data used in this report and raw data files generated from Agilent microarray systems as.gz files.

## Technical Validation

### miRNA microarray experiments

In this study, microarray experiments were performed according to the manufacturer’s instructions, and the quality of each experiment was assessed in QC reports generated from the Agilent microarray systems.

### Possible effects of peripheral blood cell-derived miRNAs

**Figure 1:**
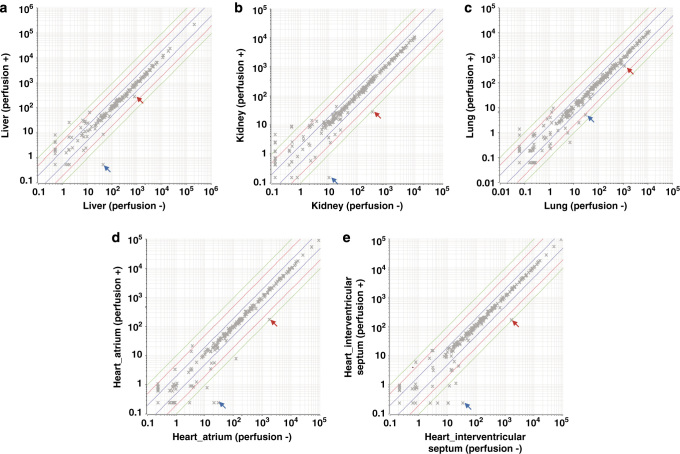
Effect of circulating blood on miRNA expression in organs. Arrows indicate blood specific miRNAs; red arrows: miR-451, blue arrows: miR-144. Horizontal axis: Perfusion (-), vertical axis: Perfusion (+). Correlation coefficient (*R*^*2*^); (**a**) Liver: 0.9998, (**b**) Kidney: 0.9960, (**c**) Lung: 0.9956, (**d**) Heart, atrium: 0.9988, (**e**) Heart, interventricular septum: 0.9994.

To evaluate the possible effects of peripheral blood cell-derived miRNAs on the miRNA expression profiles of organs, two different sampling conditions, with or without perfusion, were compared to assess effects on miRNA expression profiles in four organs (heart, kidney, liver and lung). As shown in Figure 1, blood-cell–specific miRNAs (e.g., miR-451 and miR-144)^21^ were clearly evident in nonperfused samples, but not in perfused samples. On the other hand, for each organ, miRNA profiles for perfused samples were highly correlated with those for nonperfused samples (*R*^2^>0.995). These data support the hypothesis that there are no distinct differences in miRNA profiles between these major organs and peripheral blood cells with some exceptions, such as miR-451 and miR-144, and that expression profiles for the majority of miRNAs were not markedly affected by remaining peripheral blood. Moreover, our data showed that miR-451 and miR-144 were specifically expressed in hematopoietic tissues e.g., spleen and bone marrow (refer to the original dataset for details). Therefore, microarray data obtained from nonperfused samples were analyzed in combination with those from perfused samples when generating the final comprehensive dataset.

### Principal component analysis (PCA)

**Figure 2:**
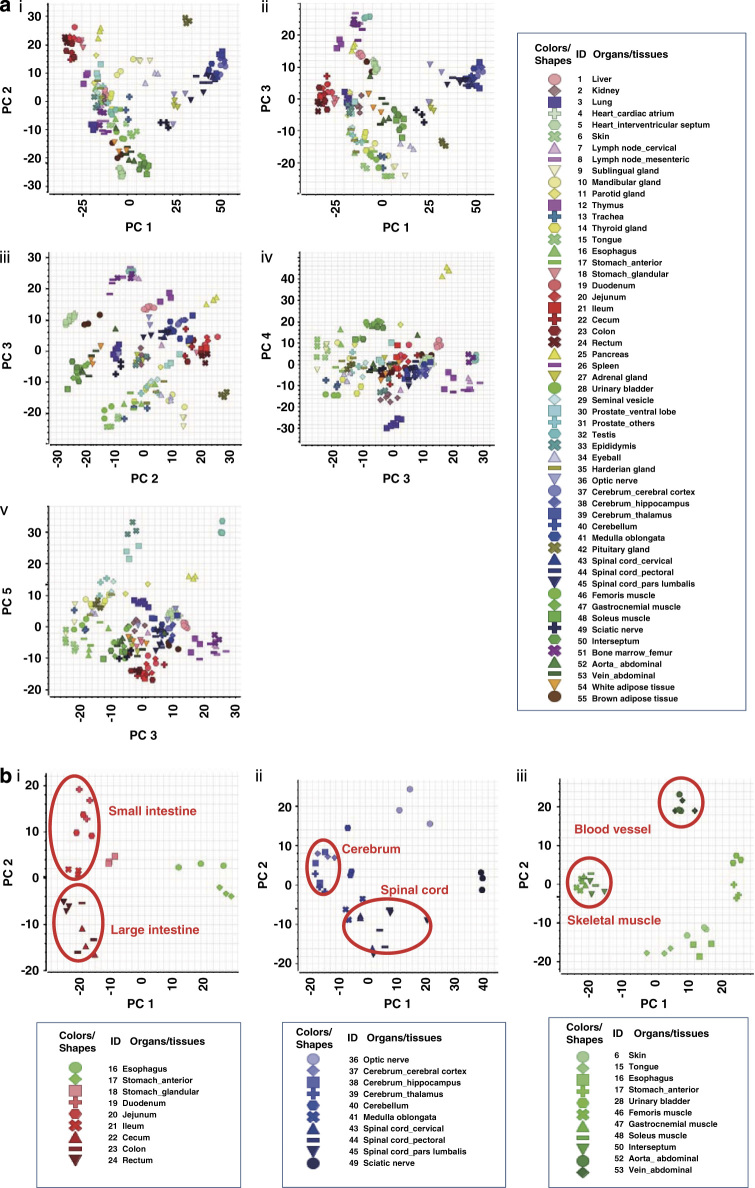
Principal component analysis (PCA). (**a**) PCA for all organ miRNAs. Spot colors represent broad types of organs; blue: nervous system, red: intestines, green: smooth or skeletal muscles, pink: lymphoid or hematopoietic system, light blue: reproductive system. Eigenvalues for each component: 29.1% (PC1), 11.4% (PC2); 8.1% (PC3), 6.7% (PC4), 5.0% (PC5). (**b**) PCA for digestive organs, nervous system or muscles. Eigenvalues for each component: (i) 37.2% (PC1), 10.8% (PC2); (ii) 35.7% (PC1), 13.2% (PC2); (iii) 31.0% (PC1), 16.4% (PC2).

PCA was performed to comprehensively compare miRNA expression profiles among all organs in the entire experimental dataset (Figure 2a). Along the principal component (PC) 1 axis, which accounts for 29.1% of all variation, the clusters of signals from nervous system (brain, spinal cord, optic nerve and sciatic nerve; shown in blue colors), pituitary gland and adrenal gland samples were clearly separated from intestine samples (shown in red), whereas along the PC2 axis (11.4%), muscle (shown in green) samples were clearly separated from nervous system and intestine samples. Along the PC3 axis (8.1%), the clusters of signals from lymph and hematopoietic tissue (shown in pink) samples were clearly separated from those of other samples. Similarly, along the PC4 axis (6.7%) and PC5 axis (5.0%), the clusters of signals from pancreas and lung samples, and those from reproductive organ samples, were clearly separated from others. A tendency towards densely clustered signals from triplicate samples of the same organ was observed in PCA results. Additional information and eigenvalues of PC1 to PC5 axes are summarized in Tables 2 and 3 (available online only).

Furthermore, miRNA microarray data obtained from digestive organs, the nervous system and skeletal/smooth muscles were extracted from the whole dataset and subjected to system-specific PCA (Figure 2b). In the intestinal-specific subanalysis, clusters of signals from upper digestive organ (esophagus and stomach) samples were clearly separated from those of lower digestive organs (small and large intestine) along PC1 (37.2%), whereas along the PC2 axis (10.8%), those from small and large intestine samples were further divided into each organ by functional anatomy of the digestive system. In the nervous system subanalysis, a clear separation of clusters of signals were observed along both PC1 (35.7%) and PC2 (13.2%); those from cerebrum samples were clearly separated from ischial nerve samples along PC1, and those from the spinal cord were separated from optic nerve samples along PC2 (13.2%). Skeletal/smooth muscles were separated into four clusters: skeletal muscles, smooth muscles (blood vessel), smooth muscles (bladder and stomach) and others (esophagus, tongue and skin) (31.0% for PC1 and 16.4% for PC2). Overall, PCA successfully showed the similarity in gene expression profiles among functionally similar organs. These results provided evidence that the microarray data were of high enough quality to assess similarities and differences among miRNA profiles from different organs and tissues. In addition, the global miRNA expression profiles for these 55 normal organs included high-quality, reliable data that reflected the biological function of each organ.

### Organ specific miRNA selection

**Figure 3:**
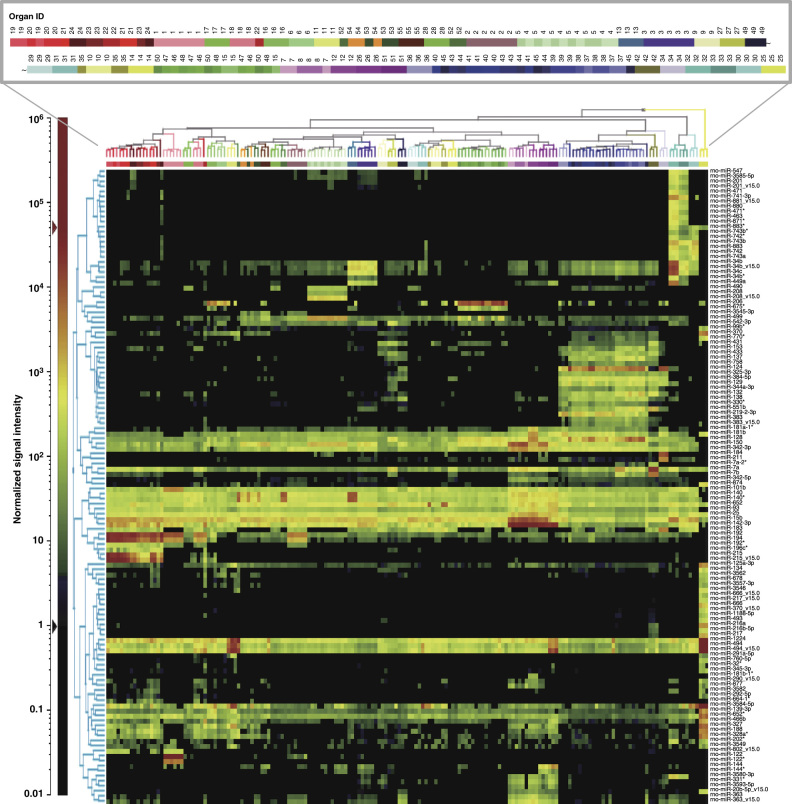
Two-dimensional hierarchical clustering analysis based on the expression profiles of the 128 filtered probes. Horizontal: individual organs, vertical: miRNAs. In this analysis, expression profiles for the 128 probes filtered by model-based clustering were used for clustering.

**Figure 4:**
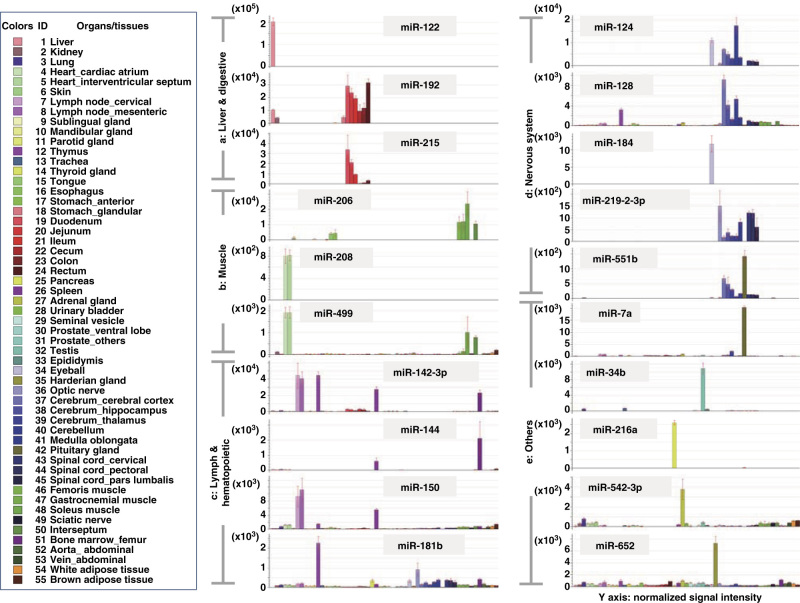
Expression profiles of organ-specific miRNAs identified in this study. Horizontal: individual organs, vertical: normalized signal intensity. Each bar represents mean±S.D. (Organ ID 1–5: *N*=6, organ ID 6–55: *N*=3).

To identify organ-specific miRNAs, all probes with any detectable signal were further filtered by a statistical analysis with criteria described in the Methods section; 296 probes met these criteria and were further analyzed using model-based clustering. In the model-based clustering, 128 probes were identified as miRNAs that were specifically expressed in one or more organs; further details on the statistical parameters calculated are available in Table 4 (available online only). Figure 3 shows a 2-dimensional hierarchical clustering diagram of groups of organs; the clustering was based on the expression profiles of the 128 filtered probes. Triplicate samples of the same organ were densely clustered; moreover, functionally similar organs were closely clustered to each other. Among these clusters, several miRNAs that were specifically expressed in specific organ(s) were successfully identified. Expression profiles of several representative organ-specific miRNAs are summarized (Figure 4 and Table 5 (available online only)). As shown in Table 5, more than half of these miRNAs were expressed in the corresponding human organs/tissues^22–31,22–31,22–31,22–31,22–31,22–31,22–31,22–31,22–31,22–31^.

### Technical validity and limitation of microarray analysis

Microarray technology has become a crucial tool for large-scale and high-throughput measurement. Non-specific hybridization or cross-hybridization is a common concern when interpreting microarrays, particularly with closely related gene family members having highly similar sequences. However, in such cases cross-hybridization is unavoidable since designing appropriate, highly specific, probes is not easy. Advances in microarray technology have successfully reduced, but not completely eliminated, cross-hybridization between the same subfamilies of miRNAs^32^. Therefore, attention should be paid to interpreting data from genes with highly similar sequences particularly in a microarray dataset similar to the present data. Recently, RNA-sequencing (RNA-seq) technology has emerged as a powerful tool for transcriptomics. This new technology is expected to be a useful tool for miRNA expression analysis.

## Usage Notes

Since a large amount of numerical data is produced with microarray analysis, sophisticated bioinformatics approaches are required to analyze and interpret the results. A great number of statistical algorithms for filtering differentially expressed genes are available through the BioConductor project website. In addition, there are several software packages available that combine visualization with statistical analysis, such as Genedata Expressionist (Genedata), GeneSpring (Agilent Technology), Spotfire DecisionSite (Spotfire), TIBCO Spotfire (TIBCO Software Inc.) and ArrayTrack (Tong *et al.*,^33^).

## Additional information

Tables 1, 2, 3, 4, 5 are only available in the online version of this paper.

**How to cite this article:** Minami, K. *et al.* miRNA expression atlas in male rat. *Sci. Data* 1:140005 doi: 10.1038/sdata.2014.5 (2014).

## Supplementary Material



## Figures and Tables

**Table 1:** List of organs/tissues evaluated in this study

**Organ ID**	**Organ name**	**Organ ID**	**Organ name**
1	Liver*	29	Seminal vesicle
2	Kidney†	30	Prostate_ventral lobe
3	Lung	31	Prostate_others
4	Heart_cardiac atrium	32	Testis
5	Heart_interventricular septum	33	Epididymis
6	Skin	34	Eyeball
7	Lymph node_cervical	35	Harderian gland
8	Lymph node_mesenteric	36	Optic nerve
9	Sublingual gland	37	Cerebrum_cerebral cortex‡
10	Mandibular gland	38	Cerebrum_hippocampus‡
11	Parotid gland	39	Cerebrum_thalamus‡
12	Thymus	40	Cerebellum
13	Trachea	41	Medulla oblongata
14	Thyroid gland	42	Pituitary gland
15	Tongue	43	Spinal cord_cervical
16	Esophagus	44	Spinal cord_pectoral
17	Stomach_anterior	45	Spinal cord_pars lumbalis
18	Stomach_glandular	46	Femoris muscle
19	Duodenum§	47	Gastrocnemial muscle
20	Jejunum§	48	Soleus muscle
21	Ileum§	49	Sciatic nerve
22	Cecum§	50	Interseptum
23	Colon§	51	Bone marrow_femur
24	Rectum§	52	Aorta_ abdominal
25	Pancreas	53	Vein_ abdominal
26	Spleen	54	White adipose tissue
27	Adrenal gland	55	Brown adipose tissue
28	Urinary bladder		

*Left lateral lobe.

^†^Whole kidney containing cortex, medulla, and papilla.

^‡^Each cerebrum region, including the cortex, hippocampus and thalamus, was macroscopically collected from the sagittal section of the cerebrum.

^§^Scrapped mucosal epithelial tissue.

**Table 2:** Individual sample IDs and additional information

**Sample ID** *	**Rat number**	**Tissue** †	**Perfused? y/n**	**Organ type category**
GSM1275420	1	No.1_liver	n	others
GSM1275421	1	No.1_kidney	n	others
GSM1275422	1	No.1_lung	n	others
GSM1275423	1	No.1_heart_atrium	n	smooth or skeletal muscles
GSM1275424	1	No.1_heart_interventricular septum	n	smooth or skeletal muscles
GSM1275425	2	No.2_liver	n	others
GSM1275426	2	No.2_kidney	n	others
GSM1275427	2	No.2_lung	n	others
GSM1275428	2	No.2_heart_atrium	n	smooth or skeletal muscles
GSM1275429	2	No.2_heart_interventricular septum	n	smooth or skeletal muscles
GSM1275430	3	No.3_liver	n	others
GSM1275431	3	No.3_kidney	n	others
GSM1275432	3	No.3_lung	n	others
GSM1275433	3	No.3_heart_atrium	n	smooth or skeletal muscles
GSM1275434	3	No.3_heart_interventricular septum	n	smooth or skeletal muscles
GSM1275435	4	No.4_liver	y	others
GSM1275436	4	No.4_kidney	y	others
GSM1275437	4	No.4_lung	y	others
GSM1275438	4	No.4_heart_atrium	y	smooth or skeletal muscles
GSM1275439	4	No.4_heart_interventricular septum	y	smooth or skeletal muscles
GSM1275440	4	No.4_skin	y	smooth or skeletal muscles
GSM1275441	4	No.4_lymph node—cervical	y	lymphoid or hematopoietic system
GSM1275442	4	No.4_lymph node—mesenteric	y	lymphoid or hematopoietic system
GSM1275443	4	No.4_sublingual gland	y	gland
GSM1275444	4	No.4_mandibular gland	y	gland
GSM1275445	4	No.4_parotid gland	y	gland
GSM1275446	4	No.4_thymus	y	lymphoid or hematopoietic system
GSM1275447	4	No.4_trachea	y	others
GSM1275448	4	No.4_thyroid	y	gland
GSM1275449	4	No.4_tongue	y	smooth or skeletal muscles
GSM1275450	4	No.4_esophagus	y	smooth or skeletal muscles
GSM1275451	4	No.4_stomach_anterior	y	smooth or skeletal muscles
GSM1275452	4	No.4_stomach_glandular	y	smooth or skeletal muscles
GSM1275453	4	No.4_duodenum	y	intestines (mucosal epithelial tissue)
GSM1275454	4	No.4_jejunum	y	intestines (mucosal epithelial tissue)
GSM1275455	4	No.4_ileum	y	intestines (mucosal epithelial tissue)
GSM1275456	4	No.4_cecum	y	intestines (mucosal epithelial tissue)
GSM1275457	4	No.4_colon	y	intestines (mucosal epithelial tissue)
GSM1275458	4	No.4_rectum	y	intestines (mucosal epithelial tissue)
GSM1275459	4	No.4_pancreas	y	gland
GSM1275460	4	No.4_spleen	y	lymphoid or hematopoietic system
GSM1275461	4	No.4_adrenal gland	y	gland
GSM1275462	4	No.4_bladder	y	smooth or skeletal muscles
GSM1275463	4	No.4_seminal vesicle	y	reproductive system
GSM1275464	4	No.4_prostate_ventral	y	reproductive system
GSM1275465	5	No.5_liver	y	others
GSM1275466	5	No.5_kidney	y	others
GSM1275467	5	No.5_lung	y	others
GSM1275468	5	No.5_heart_atrium	y	smooth or skeletal muscles
GSM1275469	5	No.5_heart_interventricular septum	y	smooth or skeletal muscles
GSM1275470	5	No.5_skin	y	smooth or skeletal muscles
GSM1275471	5	No.5_lymph node—cervical	y	lymphoid or hematopoietic system
GSM1275472	5	No.5_lymph node—mesenteric	y	lymphoid or hematopoietic system
GSM1275473	5	No.5_sublingual gland	y	gland
GSM1275474	5	No.5_mandibular gland	y	gland
GSM1275475	5	No.5_parotid gland	y	gland
GSM1275476	5	No.5_thymus	y	lymphoid or hematopoietic system
GSM1275477	5	No.5_trachea	y	others
GSM1275478	5	No.5_thyroid	y	gland
GSM1275479	5	No.5_tongue	y	smooth or skeletal muscles
GSM1275480	5	No.5_esophagus	y	smooth or skeletal muscles
GSM1275481	5	No.5_stomach_anterior	y	smooth or skeletal muscles
GSM1275482	5	No.5_stomach_glandular	y	smooth or skeletal muscles
GSM1275483	5	No.5_duodenum	y	intestines (mucosal epithelial tissue)
GSM1275484	5	No.5_jejunum	y	intestines (mucosal epithelial tissue)
GSM1275485	5	No.5_ileum	y	intestines (mucosal epithelial tissue)
GSM1275486	5	No.5_cecum	y	intestines (mucosal epithelial tissue)
GSM1275487	5	No.5_colon	y	intestines (mucosal epithelial tissue)
GSM1275488	5	No.5_rectum	y	intestines (mucosal epithelial tissue)
GSM1275489	5	No.5_pancreas	y	gland
GSM1275490	5	No.5_spleen	y	lymphoid or hematopoietic system
GSM1275491	5	No.5_adrenal gland	y	gland
GSM1275492	5	No.5_bladder	y	smooth or skeletal muscles
GSM1275493	5	No.5_seminal vesicle	y	reproductive system
GSM1275494	5	No.5_prostate_ventral	y	reproductive system
GSM1275495	5	No.5_prostate_other	y	reproductive system
GSM1275496	6	No.6_liver	y	others
GSM1275497	6	No.6_kidney	y	others
GSM1275498	6	No.6_lung	y	others
GSM1275499	6	No.6_heart_atrium	y	smooth or skeletal muscles
GSM1275500	6	No.6_heart_interventricular septum	y	smooth or skeletal muscles
GSM1275501	6	No.6_skin	y	smooth or skeletal muscles
GSM1275502	6	No.6_lymph node—cervical	y	lymphoid or hematopoietic system
GSM1275503	6	No.6_lymph node—mesenteric	y	lymphoid or hematopoietic system
GSM1275504	6	No.6_sublingual gland	y	gland
GSM1275505	6	No.6_mandibular gland	y	gland
GSM1275506	6	No.6_parotid gland	y	gland
GSM1275507	6	No.6_thymus	y	lymphoid or hematopoietic system
GSM1275508	6	No.6_trachea	y	others
GSM1275509	6	No.6_thyroid	y	gland
GSM1275510	6	No.6_tongue	y	smooth or skeletal muscles
GSM1275511	6	No.6_esophagus	y	smooth or skeletal muscles
GSM1275512	6	No.6_stomach_anterior	y	smooth or skeletal muscles
GSM1275513	6	No.6_stomach_glandular	y	smooth or skeletal muscles
GSM1275514	6	No.6_duodenum	y	intestines (mucosal epithelial tissue)
GSM1275515	6	No.6_jejunum	y	intestines (mucosal epithelial tissue)
GSM1275516	6	No.6_ileum	y	intestines (mucosal epithelial tissue)
GSM1275517	6	No.6_cecum	y	intestines (mucosal epithelial tissue)
GSM1275518	6	No.6_colon	y	intestines (mucosal epithelial tissue)
GSM1275519	6	No.6_rectum	y	intestines (mucosal epithelial tissue)
GSM1275520	6	No.6_pancreas	y	gland
GSM1275521	6	No.6_spleen	y	lymphoid or hematopoietic system
GSM1275522	6	No.6_adrenal gland	y	gland
GSM1275523	6	No.6_bladder	y	smooth or skeletal muscles
GSM1275524	6	No.6_seminal vesicle	y	reproductive system
GSM1275525	6	No.6_prostate_ventral	y	reproductive system
GSM1275526	4	No.4_prostate_other	y	reproductive system
GSM1275527	4	No.4_testicle	y	reproductive system
GSM1275528	4	No.4_epididymis	y	reproductive system
GSM1275529	4	No.4_eyeball	y	nervous system
GSM1275530	4	No.4_Harderian gland	y	gland
GSM1275531	4	No.4_optic nerve	y	nervous system
GSM1275532	4	No.4_cerebrum_cerebral cortex	y	nervous system
GSM1275533	4	No.4_cerebrum_hippocampus	y	nervous system
GSM1275534	4	No.4_cerebrum_Thalamus	y	nervous system
GSM1275535	4	No.4_cerebellum	y	nervous system
GSM1275536	4	No.4_medulla oblongata	y	nervous system
GSM1275537	4	No.4_Pituitary gland	y	gland
GSM1275538	4	No.4_spinal cord_cervical	y	nervous system
GSM1275539	4	No.4_spinal cord_pectoral	y	nervous system
GSM1275540	4	No.4_spinal cord_pars lumbalis	y	nervous system
GSM1275541	4	No.4_femoris muscle	y	smooth or skeletal muscles
GSM1275542	4	No.4_gastrocnemial muscle	y	smooth or skeletal muscles
GSM1275543	4	No.4_musculus soleus	y	smooth or skeletal muscles
GSM1275544	4	No.4_ischial nerve	y	nervous system
GSM1275545	4	No.4_interseptum	y	smooth or skeletal muscles
GSM1275546	4	No.4_bone marrow	y	lymphoid or hematopoietic system
GSM1275547	4	No.4_aorta	y	smooth or skeletal muscles
GSM1275548	4	No.4_vein	y	smooth or skeletal muscles
GSM1275549	4	No.4_white adipose tissue	y	others
GSM1275550	4	No.4_brown adipose tissue	y	others
GSM1275551	5	No.5_testicle	y	reproductive system
GSM1275552	5	No.5_epididymis	y	reproductive system
GSM1275553	5	No.5_eyeball	y	nervous system
GSM1275554	5	No.5_Harderian gland	y	gland
GSM1275555	5	No.5_optic nerve	y	nervous system
GSM1275556	5	No.5_cerebrum_cerebral cortex	y	nervous system
GSM1275557	5	No.5_cerebrum_hippocampus	y	nervous system
GSM1275558	5	No.5_cerebrum_Thalamus	y	nervous system
GSM1275559	5	No.5_cerebellum	y	nervous system
GSM1275560	5	No.5_medulla oblongata	y	nervous system
GSM1275561	5	No.5_Pituitary gland	y	gland
GSM1275562	5	No.5_spinal cord_cervical	y	nervous system
GSM1275563	5	No.5_spinal cord_pectoral	y	nervous system
GSM1275564	5	No.5_spinal cord_pars lumbalis	y	nervous system
GSM1275565	5	No.5_femoris muscle	y	smooth or skeletal muscles
GSM1275566	5	No.5_gastrocnemial muscle	y	smooth or skeletal muscles
GSM1275567	5	No.5_musculus soleus	y	smooth or skeletal muscles
GSM1275568	5	No.5_ischial nerve	y	nervous system
GSM1275569	5	No.5_interseptum	y	smooth or skeletal muscles
GSM1275570	5	No.5_bone marrow	y	lymphoid or hematopoietic system
GSM1275571	5	No.5_aorta	y	smooth or skeletal muscles
GSM1275572	5	No.5_vein	y	smooth or skeletal muscles
GSM1275573	5	No.5_white adipose tissue	y	others
GSM1275574	5	No.5_brown adipose tissue	y	others
GSM1275575	6	No.6_prostate_other	y	reproductive system
GSM1275576	6	No.6_testicle	y	reproductive system
GSM1275577	6	No.6_epididymis	y	reproductive system
GSM1275578	6	No.6_eyeball	y	nervous system
GSM1275579	6	No.6_Harderian gland	y	gland
GSM1275580	6	No.6_optic nerve	y	nervous system
GSM1275581	6	No.6_cerebrum_cerebral cortex	y	nervous system
GSM1275582	6	No.6_cerebrum_hippocampus	y	nervous system
GSM1275583	6	No.6_cerebrum_Thalamus	y	nervous system
GSM1275584	6	No.6_cerebellum	y	nervous system
GSM1275585	6	No.6_medulla oblongata	y	nervous system
GSM1275586	6	No.6_Pituitary gland	y	gland
GSM1275587	6	No.6_spinal cord_cervical	y	nervous system
GSM1275588	6	No.6_spinal cord_pectoral	y	nervous system
GSM1275589	6	No.6_spinal cord_pars lumbalis	y	nervous system
GSM1275590	6	No.6_femoris muscle	y	smooth or skeletal muscles
GSM1275591	6	No.6_gastrocnemial muscle	y	smooth or skeletal muscles
GSM1275592	6	No.6_musculus soleus	y	smooth or skeletal muscles
GSM1275593	6	No.6_ischial nerve	y	nervous system
GSM1275594	6	No.6_interseptum	y	smooth or skeletal muscles
GSM1275595	6	No.6_bone marrow	y	lymphoid or hematopoietic system
GSM1275596	6	No.6_aorta	y	smooth or skeletal muscles
GSM1275597	6	No.6_vein	y	smooth or skeletal muscles
GSM1275598	6	No.6_white adipose tissue	y	others
GSM1275599	6	No.6_brown adipose tissue	y	others

*Individual sample IDs created by GEO.

^†^Resistered tissue names in GSE52754.

**Table 3:** Eigen values and vectors of PCAs in Figure 1

**Probe ID**	**Eigenvalue 1: 29.1%**	**Eigenvalue 2: 11.4%**	**Eigenvalue 3: 8.1%**	**Eigenvalue 4: 6.7%**	**Eigenvalue 5: 5.0%**
rno-let-7a	0.001	−0.024	−0.008	−0.013	0.003
rno-let-7a-1*	0.004	−0.015	−0.026	−0.029	−0.035
rno-let-7b	−0.002	−0.018	−0.013	−0.002	0.004
rno-let-7b*	−0.025	0.013	0.013	0.040	0.015
rno-let-7c	0.002	−0.020	−0.017	−0.007	0.009
rno-let-7c-1*	0.023	−0.008	−0.014	−0.006	0.030
rno-let-7d	0.002	−0.027	−0.005	−0.014	−0.010
rno-let-7d*	0.008	−0.036	0.001	−0.027	−0.060
rno-let-7e	0.023	−0.034	−0.020	−0.044	0.001
rno-let-7f	−0.002	−0.023	−0.005	−0.018	−0.005
rno-let-7i	0.001	−0.027	−0.008	−0.014	−0.004
rno-let-7i*	−0.015	−0.004	0.008	0.035	0.014
rno-miR-1	0.011	−0.204	−0.135	0.113	−0.015
rno-miR-1*	−0.023	0.015	0.019	0.053	0.002
rno-miR-100	0.026	−0.056	−0.018	−0.018	0.033
rno-miR-100*	−0.012	−0.006	0.011	0.037	0.014
rno-miR-101a	−0.006	−0.016	−0.008	−0.003	0.008
rno-miR-101a*	−0.014	−0.006	0.007	0.037	0.015
rno-miR-101b	−0.007	−0.012	0.011	−0.025	0.001
rno-miR-103	0.007	−0.014	−0.003	−0.054	−0.030
rno-miR-105	−0.015	−0.007	0.007	0.041	0.013
rno-miR-106b	−0.022	−0.011	0.012	−0.030	−0.026
rno-miR-106b*	−0.026	−0.007	0.041	0.012	0.002
rno-miR-107	0.004	−0.016	−0.002	−0.043	−0.026
rno-miR-10a-3p	−0.036	0.000	−0.016	−0.035	−0.018
rno-miR-10a-5p	−0.043	−0.055	0.000	−0.027	−0.038
rno-miR-10b	−0.045	−0.071	−0.039	0.007	−0.024
rno-miR-10b*	−0.006	−0.027	0.019	−0.009	−0.021
rno-miR-1188-3p	−0.019	0.004	0.014	0.038	0.012
rno-miR-1188-5p	−0.011	0.015	0.018	0.068	0.031
rno-miR-122	−0.029	−0.009	0.044	0.041	0.019
rno-miR-122*	−0.020	−0.004	0.037	0.061	0.016
rno-miR-1224	−0.028	0.016	0.027	0.078	0.011
rno-miR-124	0.131	0.069	0.066	−0.009	0.016
rno-miR-124*	0.035	0.025	0.025	0.020	0.013
rno-miR-1249	−0.062	0.040	0.063	0.080	−0.063
rno-miR-125a-3p	−0.009	0.024	0.022	0.012	−0.046
rno-miR-125a-5p	0.023	−0.039	−0.015	−0.054	−0.001
rno-miR-125b*	0.037	0.002	−0.002	0.004	0.020
rno-miR-125b-5p	0.027	−0.044	−0.023	−0.009	0.038
rno-miR-126	−0.003	−0.069	0.005	−0.019	−0.006
rno-miR-126*	0.004	−0.086	0.004	−0.047	−0.028
rno-miR-127	0.113	−0.023	−0.097	0.063	0.012
rno-miR-127*	0.079	0.033	−0.025	0.039	−0.016
rno-miR-128	0.046	−0.019	0.021	−0.028	−0.041
rno-miR-128-2*	−0.004	0.002	0.021	0.032	0.011
rno-miR-129	0.102	0.067	0.046	0.007	0.034
rno-miR-129-1*	0.068	0.058	0.025	−0.002	0.023
rno-miR-129-2*	0.116	0.079	0.036	−0.030	0.017
rno-miR-130a	−0.005	−0.040	−0.009	−0.041	−0.010
rno-miR-130b	−0.077	0.074	0.044	−0.061	−0.095
rno-miR-130b*	−0.043	0.037	0.033	0.023	−0.050
rno-miR-132	0.093	0.074	0.025	−0.029	−0.011
rno-miR-132*	−0.006	0.000	0.012	0.036	0.016
rno-miR-133a	0.001	−0.172	−0.096	0.115	−0.009
rno-miR-133a*	−0.009	−0.171	−0.092	0.157	0.000
rno-miR-133b	0.013	−0.151	−0.130	0.063	−0.005
rno-miR-134	0.002	0.038	0.003	0.085	0.018
rno-miR-134*	−0.006	0.027	0.001	0.036	−0.011
rno-miR-135a	0.070	0.073	−0.033	−0.032	0.090
rno-miR-135a*	0.004	0.017	0.007	0.035	0.044
rno-miR-135b	0.075	0.046	−0.019	−0.012	0.046
rno-miR-136	0.100	0.000	−0.099	0.052	−0.015
rno-miR-136*	0.101	0.015	−0.081	0.058	−0.037
rno-miR-137	0.094	0.058	0.032	0.003	−0.014
rno-miR-137*	0.013	0.011	0.024	0.030	0.014
rno-miR-138	0.090	0.053	0.071	−0.012	−0.009
rno-miR-138-1*	−0.011	−0.005	0.011	0.037	0.013
rno-miR-138-2*	0.045	0.024	0.035	0.021	−0.001
rno-miR-139-3p	−0.017	−0.001	−0.004	0.067	−0.001
rno-miR-139-5p	0.020	−0.035	0.020	−0.044	−0.093
rno-miR-140	−0.003	−0.030	−0.002	−0.047	−0.035
rno-miR-140*	−0.006	−0.028	0.001	−0.037	−0.033
rno-miR-141	−0.128	0.143	−0.149	−0.046	0.068
rno-miR-141*	−0.012	0.000	0.001	0.038	0.016
rno-miR-142-3p	−0.046	−0.021	0.050	−0.054	−0.056
rno-miR-142-5p	−0.090	−0.019	0.060	−0.085	−0.101
rno-miR-143	−0.024	−0.054	−0.039	−0.022	0.013
rno-miR-143*	−0.033	−0.038	−0.036	−0.024	0.037
rno-miR-144	−0.027	−0.069	0.078	0.000	0.002
rno-miR-144*	−0.019	−0.042	0.051	−0.001	0.006
rno-miR-145	−0.023	−0.072	−0.055	−0.023	0.021
rno-miR-145*	−0.016	−0.026	−0.014	0.023	0.008
rno-miR-146a	−0.024	−0.038	0.008	−0.015	−0.016
rno-miR-146b	0.009	−0.019	−0.026	−0.051	−0.021
rno-miR-147	−0.041	0.037	0.015	0.034	−0.052
rno-miR-148b-3p	−0.002	−0.007	−0.009	−0.041	−0.005
rno-miR-148b-5p	−0.011	0.004	0.003	0.015	0.015
rno-miR-150	0.002	−0.065	0.038	−0.049	−0.046
rno-miR-150*	−0.060	0.035	0.056	0.051	−0.101
rno-miR-151	0.003	−0.032	−0.011	−0.042	−0.009
rno-miR-151*	−0.001	−0.008	0.011	−0.009	0.013
rno-miR-152	−0.022	−0.029	−0.045	0.012	0.016
rno-miR-153	0.066	0.090	0.011	−0.014	−0.015
rno-miR-154	0.099	−0.001	−0.104	0.033	−0.042
rno-miR-154*	0.069	0.058	−0.012	0.018	0.007
rno-miR-15b	−0.027	−0.012	0.015	−0.026	−0.014
rno-miR-15b*	−0.037	0.018	0.042	−0.012	−0.032
rno-miR-16	−0.015	−0.024	0.011	−0.022	−0.021
rno-miR-17-1-3p	−0.039	0.003	0.057	−0.005	−0.032
rno-miR-17-5p	−0.031	−0.010	0.022	−0.038	−0.038
rno-miR-181a	0.012	−0.013	0.027	−0.016	−0.010
rno-miR-181a-1*	0.040	−0.038	0.053	−0.067	−0.034
rno-miR-181b	0.022	−0.016	0.019	−0.016	−0.024
rno-miR-181b-1*	−0.018	−0.003	0.022	0.032	0.009
rno-miR-181c	0.006	−0.019	−0.009	−0.053	−0.032
rno-miR-181d	0.005	−0.007	0.005	−0.022	−0.022
rno-miR-182	−0.042	0.081	−0.127	−0.031	0.026
rno-miR-183	−0.040	0.115	−0.158	−0.072	0.032
rno-miR-183*	−0.024	0.033	−0.019	0.032	−0.010
rno-miR-184	−0.013	−0.016	0.006	0.010	0.030
rno-miR-185	0.012	0.000	0.003	−0.018	−0.017
rno-miR-186	−0.005	−0.009	0.008	−0.002	−0.010
rno-miR-187*	−0.015	−0.001	0.011	0.044	0.016
rno-miR-188	−0.075	0.065	0.050	0.085	−0.067
rno-miR-188*	−0.026	−0.008	0.055	0.010	−0.009
rno-miR-18a	−0.071	0.025	0.034	−0.049	−0.082
rno-miR-190	0.004	0.002	−0.003	0.032	−0.022
rno-miR-190*	−0.007	0.003	0.008	0.038	−0.003
rno-miR-191	−0.020	−0.007	0.032	0.022	−0.001
rno-miR-191*	−0.007	−0.013	0.028	−0.027	−0.041
rno-miR-192	−0.045	0.062	0.023	−0.045	−0.133
rno-miR-192*	−0.067	0.073	0.025	0.025	−0.092
rno-miR-193	−0.036	−0.042	−0.027	0.023	−0.027
rno-miR-193*	−0.024	−0.065	−0.039	0.035	−0.050
rno-miR-194	−0.045	0.095	0.039	−0.052	−0.181
rno-miR-194*	−0.054	0.056	0.018	0.026	−0.070
rno-miR-1949	−0.055	−0.039	0.009	0.001	−0.054
rno-miR-195	−0.002	−0.063	−0.018	−0.022	−0.008
rno-miR-196a	−0.032	−0.021	−0.046	0.027	−0.081
rno-miR-196b	−0.044	−0.026	−0.032	0.017	−0.061
rno-miR-196c	−0.039	−0.004	−0.044	0.033	−0.088
rno-miR-196c*	−0.051	0.060	0.011	0.036	−0.091
rno-miR-199a-3p	−0.027	−0.069	−0.048	−0.001	−0.005
rno-miR-199a-5p	−0.041	−0.080	−0.053	−0.009	−0.015
rno-miR-19a	−0.056	0.008	0.035	−0.042	−0.033
rno-miR-19b	−0.039	0.002	0.028	−0.009	−0.008
rno-miR-19b-1*	−0.025	−0.011	0.058	0.011	−0.006
rno-miR-200a	−0.130	0.157	−0.197	−0.039	0.081
rno-miR-200a*	−0.052	0.065	−0.025	0.028	0.000
rno-miR-200b	−0.134	0.158	−0.206	−0.040	0.082
rno-miR-200b*	−0.082	0.099	−0.090	0.006	0.067
rno-miR-200c	−0.098	0.118	−0.161	−0.018	0.090
rno-miR-200c_v15.0	−0.134	0.121	−0.135	−0.055	0.111
rno-miR-201	−0.028	−0.016	0.031	−0.003	0.103
rno-miR-201*	−0.017	−0.008	0.022	0.034	0.065
rno-miR-201_v15.0	−0.026	−0.014	0.028	0.006	0.094
rno-miR-202	−0.014	−0.007	0.013	0.038	0.022
rno-miR-202*	−0.029	0.034	0.027	0.071	0.021
rno-miR-202_v15.0	−0.015	−0.008	0.025	0.040	0.046
rno-miR-203	−0.090	0.046	−0.139	−0.034	−0.071
rno-miR-204	0.039	0.005	−0.051	−0.061	0.029
rno-miR-204*	0.026	0.015	0.011	0.018	0.017
rno-miR-205	−0.080	0.042	−0.210	−0.041	0.051
rno-miR-205_v15.0	−0.088	0.037	−0.216	−0.035	0.032
rno-miR-206	0.020	−0.080	−0.104	0.189	−0.035
rno-miR-207	−0.015	−0.007	0.015	0.036	0.013
rno-miR-207_v15.0	−0.015	−0.007	0.015	0.036	0.013
rno-miR-208	−0.016	−0.053	0.035	0.043	0.031
rno-miR-208*	−0.017	−0.052	0.030	0.040	0.034
rno-miR-208_v15.0	−0.017	−0.071	0.046	0.045	0.037
rno-miR-208b-3p	−0.013	−0.011	0.007	0.041	0.012
rno-miR-20a	−0.031	−0.013	0.025	−0.039	−0.037
rno-miR-20a*	−0.054	0.027	0.045	−0.023	−0.041
rno-miR-20b-3p	−0.016	−0.006	0.018	0.032	0.008
rno-miR-20b-5p	−0.031	−0.014	0.026	−0.037	−0.037
rno-miR-20b-5p_v15.0	−0.026	−0.003	0.081	0.035	−0.002
rno-miR-21	−0.038	−0.001	−0.006	−0.017	−0.007
rno-miR-21*	−0.033	0.019	0.020	0.028	−0.032
rno-miR-210	−0.003	−0.021	−0.003	−0.007	−0.012
rno-miR-211	0.023	0.009	0.012	−0.041	0.064
rno-miR-211*	−0.064	0.065	0.023	0.075	−0.077
rno-miR-212	0.014	0.082	0.040	0.065	−0.064
rno-miR-212*	0.007	0.015	0.011	0.033	0.022
rno-miR-214	−0.035	−0.077	−0.048	0.006	−0.008
rno-miR-215	−0.076	0.096	0.012	0.046	−0.133
rno-miR-215_v15.0	−0.082	0.103	0.014	0.047	−0.146
rno-miR-216a	−0.011	0.025	0.017	0.085	0.044
rno-miR-216b-5p	−0.011	0.017	0.012	0.071	0.039
rno-miR-217	−0.012	0.019	0.016	0.081	0.043
rno-miR-217_v15.0	−0.016	0.007	0.019	0.071	0.033
rno-miR-218	0.067	−0.055	−0.041	−0.072	0.057
rno-miR-219-2-3p	0.096	0.049	0.068	0.003	−0.002
rno-miR-219-5p	0.053	0.092	0.062	−0.087	−0.096
rno-miR-22	−0.005	−0.049	−0.014	0.017	−0.002
rno-miR-22*	0.006	−0.054	−0.038	−0.012	−0.028
rno-miR-221	0.000	−0.017	−0.025	−0.055	0.019
rno-miR-221*	−0.004	0.001	0.008	0.020	0.023
rno-miR-222	0.013	0.025	−0.036	−0.088	0.047
rno-miR-223	−0.025	−0.026	0.013	0.000	−0.018
rno-miR-224	−0.012	−0.004	−0.005	0.036	0.016
rno-miR-224*	−0.011	−0.003	−0.001	0.040	0.016
rno-miR-224_v15.0	−0.019	−0.110	−0.102	0.015	0.055
rno-miR-23a	−0.011	−0.046	−0.028	−0.007	−0.020
rno-miR-23a*	−0.027	0.009	0.001	0.048	−0.013
rno-miR-23b	0.001	−0.029	−0.033	−0.008	−0.008
rno-miR-24	−0.006	−0.038	−0.026	−0.007	−0.015
rno-miR-24-2*	0.002	−0.061	−0.037	−0.037	−0.045
rno-miR-25	−0.019	−0.012	0.013	−0.004	−0.006
rno-miR-26a	−0.003	−0.028	−0.005	−0.011	−0.006
rno-miR-26b	−0.012	−0.024	−0.010	−0.017	−0.008
rno-miR-26b*	−0.026	−0.014	0.020	−0.017	−0.006
rno-miR-27a	−0.017	−0.040	−0.023	−0.005	−0.032
rno-miR-27b	−0.005	−0.024	−0.026	0.004	−0.011
rno-miR-28	−0.021	−0.037	−0.019	−0.025	−0.027
rno-miR-28*	−0.016	−0.011	0.003	0.031	0.012
rno-miR-290	−0.076	−0.011	0.025	0.054	−0.011
rno-miR-290_v15.0	−0.043	0.028	0.050	0.061	−0.003
rno-miR-291a-5p	−0.021	0.008	0.011	0.074	0.032
rno-miR-292-5p	−0.031	0.025	0.016	0.063	−0.012
rno-miR-294	−0.015	−0.006	0.007	0.043	0.015
rno-miR-295	−0.017	−0.003	0.012	0.038	0.008
rno-miR-295_v15.0	−0.017	−0.003	0.011	0.038	0.008
rno-miR-296*	0.019	0.035	0.084	0.046	−0.016
rno-miR-298	0.006	0.043	0.039	0.042	0.007
rno-miR-2985	−0.015	−0.006	0.011	0.037	0.013
rno-miR-299	0.074	−0.029	−0.122	0.070	−0.017
rno-miR-299*	−0.011	0.001	0.002	0.037	0.018
rno-miR-29a	0.002	−0.012	0.002	−0.010	0.005
rno-miR-29a*	0.002	−0.019	−0.018	−0.040	−0.008
rno-miR-29b	0.003	0.009	0.005	−0.029	0.005
rno-miR-29b-1*	−0.015	−0.007	0.010	0.034	0.017
rno-miR-29b-2*	0.011	0.014	0.015	0.031	0.014
rno-miR-29c	0.004	−0.007	−0.012	−0.011	0.008
rno-miR-29c*	0.021	−0.019	−0.042	−0.023	0.004
rno-miR-300-3p	0.102	−0.001	−0.102	0.050	−0.048
rno-miR-300-5p	0.057	0.043	0.031	0.024	0.006
rno-miR-301a	0.000	−0.008	0.007	−0.054	−0.029
rno-miR-301b	−0.032	0.031	0.024	0.028	−0.034
rno-miR-3085	−0.015	−0.006	0.013	0.038	0.013
rno-miR-30a	−0.006	−0.039	−0.010	0.011	0.008
rno-miR-30a*	0.003	−0.053	−0.021	−0.018	−0.002
rno-miR-30b-3p	−0.025	−0.021	0.010	−0.002	−0.011
rno-miR-30b-5p	−0.013	−0.024	−0.009	−0.004	0.008
rno-miR-30c	−0.002	−0.035	−0.006	−0.006	−0.002
rno-miR-30c-1*	−0.006	−0.016	0.033	−0.002	−0.012
rno-miR-30c-2*	0.006	−0.037	−0.023	0.017	0.012
rno-miR-30d	−0.009	−0.026	0.003	0.012	0.005
rno-miR-30d*	−0.024	−0.006	0.017	−0.015	−0.010
rno-miR-30e	−0.014	−0.025	0.010	0.005	−0.015
rno-miR-30e*	0.001	−0.043	−0.001	−0.020	−0.020
rno-miR-31	−0.018	0.084	−0.078	−0.047	−0.028
rno-miR-31*	−0.043	0.055	−0.066	0.011	−0.044
rno-miR-32	−0.055	0.040	0.035	−0.049	−0.061
rno-miR-32*	−0.021	0.003	0.021	0.058	0.018
rno-miR-320	−0.003	−0.009	−0.021	0.007	−0.024
rno-miR-322	0.002	−0.082	−0.009	−0.033	0.003
rno-miR-322*	0.033	−0.125	−0.009	−0.034	0.020
rno-miR-323	0.091	0.058	0.028	0.004	0.001
rno-miR-324-3p	−0.004	−0.002	0.002	0.013	0.007
rno-miR-324-5p	0.014	−0.012	−0.012	−0.047	−0.013
rno-miR-325-3p	0.092	0.057	0.030	0.003	0.003
rno-miR-325-5p	0.044	0.036	0.023	0.019	0.016
rno-miR-326	0.022	0.025	0.009	−0.103	−0.048
rno-miR-326*	−0.015	−0.002	0.015	0.053	0.023
rno-miR-327	−0.079	0.063	0.018	0.066	−0.091
rno-miR-328a	0.023	−0.023	0.002	−0.041	−0.018
rno-miR-328a*	−0.050	0.046	0.027	0.102	−0.081
rno-miR-328b-3p	0.012	0.010	0.029	0.028	0.008
rno-miR-329	0.111	−0.007	−0.108	0.042	−0.037
rno-miR-33	−0.014	0.027	0.019	−0.046	−0.064
rno-miR-33*	0.008	0.046	0.038	−0.032	−0.048
rno-miR-330	0.019	0.013	0.026	0.028	0.010
rno-miR-330*	0.067	0.036	0.030	−0.026	0.010
rno-miR-331	0.016	−0.017	−0.007	−0.035	−0.011
rno-miR-331*	−0.025	−0.004	0.051	−0.016	0.004
rno-miR-335	0.068	−0.079	−0.002	−0.020	−0.038
rno-miR-337	0.081	−0.012	−0.092	0.066	−0.045
rno-miR-337*	0.070	0.003	−0.050	0.067	−0.029
rno-miR-338	0.060	−0.037	0.008	−0.037	−0.022
rno-miR-338*	0.052	0.020	0.039	0.018	−0.006
rno-miR-339-3p	−0.013	−0.010	0.021	0.002	0.004
rno-miR-339-5p	−0.014	−0.008	0.012	0.036	0.013
rno-miR-340-3p	0.032	−0.011	0.014	−0.083	0.016
rno-miR-340-5p	0.028	0.060	0.046	−0.109	0.011
rno-miR-341	0.095	0.041	−0.047	0.064	−0.033
rno-miR-342-3p	0.014	−0.016	0.036	−0.075	−0.016
rno-miR-342-5p	0.021	0.019	0.081	−0.079	0.027
rno-miR-344a-3p	0.083	0.056	0.036	0.009	0.008
rno-miR-344b-2-3p	0.019	0.020	0.022	0.029	0.019
rno-miR-344b-5p	0.023	0.026	0.020	0.029	0.021
rno-miR-345-3p	−0.024	0.016	0.018	0.072	0.006
rno-miR-345-5p	−0.003	−0.017	0.000	0.006	0.002
rno-miR-346	0.029	0.019	0.032	0.026	0.008
rno-miR-347	−0.045	0.011	0.015	0.039	−0.034
rno-miR-34a	0.023	−0.036	0.024	−0.007	0.027
rno-miR-34a*	0.022	0.000	0.039	−0.008	0.021
rno-miR-34b	0.032	−0.013	0.057	−0.110	0.122
rno-miR-34b*	−0.020	−0.014	0.020	−0.016	0.106
rno-miR-34b_v15.0	0.034	0.006	0.061	−0.121	0.129
rno-miR-34c	0.041	−0.003	0.066	−0.109	0.144
rno-miR-34c*	−0.018	−0.013	0.017	−0.003	0.066
rno-miR-350	0.034	−0.053	0.059	0.003	−0.004
rno-miR-351	−0.012	−0.016	0.006	0.011	0.013
rno-miR-351*	−0.024	0.002	0.042	0.039	0.025
rno-miR-352	0.005	−0.035	−0.014	−0.035	−0.021
rno-miR-3541	−0.015	−0.007	0.016	0.035	0.012
rno-miR-3544	−0.032	0.016	0.022	0.048	0.003
rno-miR-3545-3p	−0.019	−0.032	0.008	0.017	−0.003
rno-miR-3546	−0.018	0.004	0.021	0.081	0.035
rno-miR-3547	−0.032	0.012	0.020	0.052	−0.001
rno-miR-3549	−0.037	0.040	0.036	0.087	−0.012
rno-miR-3550	−0.015	−0.007	0.015	0.036	0.013
rno-miR-3551-3p	−0.016	−0.004	0.009	0.043	0.029
rno-miR-3554	−0.015	−0.004	0.012	0.046	0.019
rno-miR-3557-3p	−0.027	0.010	0.011	0.081	−0.004
rno-miR-3558-3p	−0.029	0.018	0.011	−0.029	0.081
rno-miR-3559-5p	−0.064	0.046	0.017	−0.067	−0.063
rno-miR-3562	−0.029	0.020	0.010	0.093	−0.011
rno-miR-3563-3p	0.001	0.007	−0.027	0.041	0.008
rno-miR-3563-5p	−0.009	−0.001	−0.001	0.037	0.013
rno-miR-3564	−0.059	0.061	0.006	0.087	−0.089
rno-miR-3568	−0.015	−0.005	0.011	0.038	0.013
rno-miR-3572	−0.015	−0.007	0.014	0.036	0.013
rno-miR-3573-3p	−0.017	−0.002	0.014	0.039	0.010
rno-miR-3573-5p	−0.016	−0.005	0.011	0.039	0.012
rno-miR-3580-3p	−0.032	−0.012	0.082	−0.014	0.080
rno-miR-3582	−0.031	0.019	0.035	0.058	0.013
rno-miR-3584-3p	−0.015	−0.007	0.015	0.036	0.013
rno-miR-3584-5p	−0.052	0.033	−0.006	0.084	0.027
rno-miR-3585-5p	−0.033	−0.039	0.041	−0.004	0.116
rno-miR-3588	0.003	−0.013	0.029	−0.001	0.057
rno-miR-3593-3p	−0.079	0.049	0.044	0.067	−0.074
rno-miR-3593-5p	−0.024	−0.006	0.060	0.001	−0.008
rno-miR-3594-5p	−0.021	0.005	0.013	0.042	−0.005
rno-miR-361	0.008	−0.015	0.013	−0.040	−0.032
rno-miR-361*	0.006	0.004	0.035	0.025	0.001
rno-miR-362	−0.041	−0.008	−0.016	0.001	−0.107
rno-miR-362*	−0.022	−0.025	−0.010	−0.018	−0.047
rno-miR-363	−0.037	−0.012	0.086	−0.060	0.002
rno-miR-363_v15.0	−0.043	0.002	0.081	−0.088	−0.012
rno-miR-365	−0.013	−0.046	−0.027	0.013	−0.009
rno-miR-369-3p	0.048	0.040	0.016	0.023	0.007
rno-miR-369-5p	0.105	0.011	−0.071	0.058	−0.040
rno-miR-370	0.032	0.055	0.010	0.116	−0.036
rno-miR-370_v15.0	−0.013	0.011	0.015	0.073	0.033
rno-miR-374	0.003	−0.024	−0.023	−0.061	−0.032
rno-miR-375	−0.070	0.162	−0.088	−0.002	0.099
rno-miR-376a	0.108	0.031	−0.067	0.005	−0.027
rno-miR-376a*	0.044	0.038	0.014	0.025	0.010
rno-miR-376b-3p	0.090	0.067	−0.020	0.002	0.006
rno-miR-376b-5p	0.078	0.021	−0.048	0.042	−0.021
rno-miR-376c	0.074	−0.006	−0.090	0.057	−0.042
rno-miR-377	−0.002	0.009	−0.006	0.037	0.014
rno-miR-377_v15.0	0.039	0.035	−0.012	0.033	0.007
rno-miR-378	−0.029	−0.040	−0.014	0.044	−0.057
rno-miR-378*	−0.031	−0.072	−0.030	0.031	−0.097
rno-miR-379	0.103	−0.007	−0.104	0.039	−0.021
rno-miR-379*	0.075	0.024	−0.022	0.029	−0.021
rno-miR-380	0.005	0.006	0.015	0.035	0.012
rno-miR-380*	0.082	0.053	0.027	0.008	−0.005
rno-miR-381	0.096	−0.003	−0.091	0.070	−0.050
rno-miR-381*	0.029	0.008	−0.009	0.052	−0.004
rno-miR-381_v15.0	−0.011	0.000	0.003	0.037	0.018
rno-miR-382	0.102	0.011	−0.074	0.041	−0.040
rno-miR-382*	0.071	0.053	0.007	0.018	−0.001
rno-miR-383	0.072	0.053	0.037	0.008	0.005
rno-miR-383_v15.0	0.065	0.051	0.043	0.020	0.006
rno-miR-384-3p	0.057	0.042	0.028	0.014	0.014
rno-miR-384-5p	0.112	0.065	0.023	−0.013	0.009
rno-miR-409-3p	−0.012	−0.002	0.011	0.036	0.013
rno-miR-409-5p	0.079	0.032	−0.016	0.047	−0.021
rno-miR-409-5p_v15.0	0.065	0.040	0.005	0.032	−0.004
rno-miR-410	0.111	0.022	−0.046	0.057	−0.035
rno-miR-411	0.090	0.005	−0.086	0.056	−0.040
rno-miR-411*	0.102	−0.009	−0.104	0.041	−0.037
rno-miR-412*	−0.003	0.001	0.016	0.035	0.016
rno-miR-421*	0.046	0.015	−0.003	−0.075	−0.004
rno-miR-423	−0.017	−0.012	0.023	0.028	0.011
rno-miR-423*	−0.016	−0.004	0.016	0.009	−0.016
rno-miR-425	−0.015	−0.003	0.020	−0.049	−0.029
rno-miR-425*	−0.015	−0.008	0.017	0.034	0.011
rno-miR-429	−0.122	0.146	−0.190	−0.041	0.071
rno-miR-431	0.075	0.031	−0.044	0.061	−0.019
rno-miR-433	0.101	0.049	0.013	0.027	−0.016
rno-miR-433*	0.067	0.041	0.027	0.020	−0.004
rno-miR-434	0.121	−0.021	−0.105	0.054	0.012
rno-miR-434*	0.087	0.036	−0.026	0.037	−0.017
rno-miR-448	0.014	0.010	0.023	0.028	0.010
rno-miR-448*	0.012	0.010	0.023	0.030	0.011
rno-miR-448_v15.0	−0.010	−0.004	0.011	0.036	0.015
rno-miR-449a	−0.045	−0.003	0.055	−0.057	0.073
rno-miR-449c-5p	−0.017	−0.010	0.005	0.017	0.021
rno-miR-450a	0.019	−0.103	−0.002	−0.036	0.031
rno-miR-450a*	−0.011	−0.007	0.009	0.037	0.011
rno-miR-450a_v15.0	−0.004	−0.119	−0.008	−0.025	−0.001
rno-miR-451	−0.034	−0.131	0.032	−0.025	−0.055
rno-miR-455	−0.017	−0.018	0.004	−0.015	−0.003
rno-miR-455*	0.021	−0.045	−0.007	−0.068	−0.059
rno-miR-463	−0.021	−0.007	0.030	0.029	0.099
rno-miR-463*	−0.015	−0.008	0.017	0.037	0.036
rno-miR-465	−0.015	−0.005	0.011	0.038	0.013
rno-miR-465*	−0.016	−0.008	0.022	0.037	0.047
rno-miR-466b	−0.033	0.018	0.024	0.043	−0.001
rno-miR-466b-1*	−0.015	−0.005	0.011	0.043	−0.011
rno-miR-466b-2*	−0.012	−0.010	0.075	0.001	−0.031
rno-miR-466c*	−0.018	−0.006	0.008	0.040	0.001
rno-miR-471	−0.023	−0.004	0.030	0.029	0.092
rno-miR-471*	−0.021	−0.007	0.029	0.030	0.094
rno-miR-483	0.008	−0.020	0.024	0.076	0.003
rno-miR-483*	−0.056	0.054	0.009	0.101	−0.121
rno-miR-484	−0.001	−0.015	−0.004	−0.034	−0.077
rno-miR-485	0.073	0.051	0.014	0.019	0.001
rno-miR-487b	0.107	0.008	−0.069	0.054	−0.047
rno-miR-487b_v15.0	0.070	0.056	0.013	0.027	−0.024
rno-miR-488	0.050	0.037	0.034	0.015	0.021
rno-miR-489	−0.008	−0.001	0.012	0.029	0.018
rno-miR-490	0.026	−0.036	0.028	0.008	0.033
rno-miR-490*	0.030	−0.013	0.030	0.020	0.028
rno-miR-493	−0.013	0.014	0.008	0.072	0.035
rno-miR-493*	−0.010	0.000	−0.004	0.040	0.016
rno-miR-494	−0.025	0.015	0.011	0.077	0.022
rno-miR-494_v15.0	−0.028	0.016	0.021	0.081	0.012
rno-miR-495	0.109	0.025	−0.054	0.054	−0.033
rno-miR-496	0.049	0.032	0.028	0.021	0.001
rno-miR-496_v15.0	0.077	0.048	0.015	0.012	−0.007
rno-miR-497	−0.005	−0.055	−0.010	−0.007	−0.001
rno-miR-499	0.017	−0.101	−0.048	−0.017	0.010
rno-miR-500	−0.014	−0.009	0.004	0.007	−0.006
rno-miR-501*	−0.015	−0.004	0.012	0.045	0.019
rno-miR-503	−0.003	−0.117	−0.011	−0.002	−0.019
rno-miR-505	0.002	−0.015	0.013	−0.051	−0.036
rno-miR-505*	0.029	0.010	0.027	−0.052	−0.009
rno-miR-511*	−0.040	−0.108	0.010	0.011	−0.004
rno-miR-532-3p	−0.005	−0.027	−0.008	−0.023	−0.040
rno-miR-532-5p	−0.014	−0.030	0.000	−0.013	−0.051
rno-miR-539	0.082	0.009	−0.054	0.060	−0.024
rno-miR-540	−0.011	0.000	0.005	0.037	0.019
rno-miR-540*	−0.012	−0.002	0.007	0.037	0.018
rno-miR-540_v15.0	−0.005	0.008	0.001	0.036	0.022
rno-miR-541	0.075	0.031	−0.023	0.042	−0.018
rno-miR-541*	−0.006	0.005	−0.008	0.040	0.014
rno-miR-542-3p	0.027	−0.114	0.017	−0.037	0.010
rno-miR-542-5p	0.040	−0.119	−0.002	−0.026	0.015
rno-miR-543*	0.092	0.052	0.006	0.019	−0.007
rno-miR-543_v15.0	0.033	0.027	0.020	0.025	0.009
rno-miR-547	−0.034	−0.033	0.037	−0.007	0.121
rno-miR-547*	−0.016	−0.008	0.025	0.039	0.052
rno-miR-547_v15.0	−0.018	−0.009	0.024	0.033	0.074
rno-miR-551b	0.091	0.061	0.037	−0.042	0.023
rno-miR-582	0.010	0.075	−0.050	−0.067	−0.048
rno-miR-582*	0.007	0.060	0.027	0.048	−0.014
rno-miR-592	0.038	0.031	0.025	0.022	0.014
rno-miR-598-3p	0.082	0.039	−0.022	−0.080	0.004
rno-miR-652	−0.009	−0.009	0.005	−0.023	0.008
rno-miR-652*	−0.034	0.005	0.009	0.053	0.004
rno-miR-653	−0.007	0.001	0.012	0.024	0.018
rno-miR-664	0.000	−0.008	0.007	0.010	0.007
rno-miR-664-1*	−0.041	0.036	0.012	0.037	−0.053
rno-miR-664-2*	0.016	0.012	0.018	0.035	0.008
rno-miR-665	0.019	0.023	0.013	0.029	0.016
rno-miR-665_v15.0	−0.004	0.011	−0.001	0.039	0.016
rno-miR-666	−0.017	0.007	0.023	0.080	0.036
rno-miR-666_v15.0	−0.018	0.005	0.020	0.069	0.026
rno-miR-667	0.052	0.060	0.014	0.031	−0.056
rno-miR-667*	−0.020	−0.001	0.005	0.046	−0.003
rno-miR-667_v15.0	0.029	0.023	0.029	0.024	0.011
rno-miR-668	−0.015	−0.007	0.014	0.036	0.013
rno-miR-671	−0.015	−0.007	0.014	0.036	0.013
rno-miR-672	0.026	0.071	−0.038	−0.056	0.178
rno-miR-674-3p	0.013	−0.023	−0.006	−0.035	−0.059
rno-miR-674-5p	0.019	−0.004	0.032	−0.033	−0.010
rno-miR-675*	−0.006	−0.063	−0.055	0.111	−0.029
rno-miR-678	−0.024	0.011	0.016	0.080	0.011
rno-miR-702-3p	−0.014	−0.006	0.011	0.036	0.013
rno-miR-708	−0.014	−0.007	0.008	0.040	0.014
rno-miR-711	−0.017	−0.003	0.011	0.038	0.008
rno-miR-741-3p	−0.026	−0.002	0.033	0.029	0.118
rno-miR-742	−0.026	−0.001	0.024	0.033	0.130
rno-miR-742*	−0.023	−0.002	0.025	0.034	0.112
rno-miR-743a	−0.025	−0.001	0.023	0.033	0.124
rno-miR-743a*	−0.019	−0.007	0.023	0.034	0.087
rno-miR-743b	−0.027	−0.001	0.026	0.032	0.147
rno-miR-743b*	−0.021	−0.005	0.024	0.036	0.102
rno-miR-758	0.070	0.050	0.033	0.014	0.004
rno-miR-760-3p	0.000	0.018	0.072	0.035	−0.017
rno-miR-760-5p	−0.021	0.010	0.017	0.074	0.023
rno-miR-764*	0.001	0.004	0.015	0.036	0.012
rno-miR-764_v15.0	0.016	0.012	0.025	0.028	0.010
rno-miR-770*	0.050	0.054	0.038	0.061	0.022
rno-miR-7a	0.007	0.051	0.038	−0.017	−0.006
rno-miR-7a-1*	0.000	0.014	−0.001	−0.032	−0.016
rno-miR-7a-2*	0.024	0.032	0.014	0.030	0.026
rno-miR-7b	0.047	0.100	0.068	−0.032	−0.033
rno-miR-802	−0.030	0.019	0.026	0.055	−0.018
rno-miR-802_v15.0	−0.038	0.041	0.030	0.059	−0.037
rno-miR-871*	−0.021	−0.007	0.029	0.030	0.096
rno-miR-871_v15.0	−0.017	−0.008	0.021	0.033	0.066
rno-miR-872	−0.009	−0.011	0.006	−0.041	−0.013
rno-miR-872*	−0.011	−0.001	0.025	−0.025	−0.015
rno-miR-873	0.043	0.034	0.045	0.010	0.005
rno-miR-874	0.017	0.032	0.059	0.008	0.026
rno-miR-877	−0.042	0.023	0.056	0.044	−0.031
rno-miR-878	−0.015	−0.008	0.016	0.037	0.036
rno-miR-880	−0.022	−0.007	0.028	0.029	0.097
rno-miR-881	−0.015	−0.008	0.022	0.039	0.039
rno-miR-881*	−0.018	−0.009	0.024	0.033	0.075
rno-miR-881_v15.0	−0.020	−0.010	0.029	0.030	0.098
rno-miR-883	−0.026	−0.001	0.025	0.034	0.133
rno-miR-883*	−0.023	0.003	0.034	0.060	0.123
rno-miR-9	0.109	0.026	0.025	−0.053	0.091
rno-miR-9*	0.117	0.044	0.028	−0.050	0.101
rno-miR-92a	−0.029	−0.011	0.036	−0.003	−0.015
rno-miR-92a-2*	−0.019	−0.009	0.033	0.029	0.003
rno-miR-92b	0.025	0.035	0.035	0.026	0.006
rno-miR-93	−0.018	−0.014	0.010	−0.040	−0.033
rno-miR-96	−0.060	0.132	−0.147	−0.079	0.024
rno-miR-96*	−0.012	−0.004	0.003	0.035	0.019
rno-miR-98	0.007	−0.015	−0.007	−0.024	−0.014
rno-miR-99a	0.019	−0.051	−0.021	0.005	0.055
rno-miR-99a*	0.033	−0.071	−0.050	−0.010	0.053
rno-miR-99b	0.024	−0.044	−0.022	−0.056	0.004
rno-miR-99b*	0.016	0.010	−0.002	0.021	0.006

**Table 4:** Summary of statistical parameters of miRNAs calculated in this study

**Probe ID**	**Min**	**25%**	**Med**	**75%**	**Max**	**P value of Shapiro-Wilk test with Bonferroni correction**	**Minimum cluster size by model-based clustering**
rno-let-7a	1046.42	6845.00	10232.27	13716.02	31554.59	8.52E-03	NaN
rno-let-7a-1*	0.17	6.19	9.34	12.50	32.73	1.08E-13	NaN
rno-let-7b	1989.44	4032.61	5945.58	8531.73	19369.52	1	NaN
rno-let-7b*	0.05	3.49	9.43	18.50	110.78	1.00E-08	NaN
rno-let-7c	1934.18	5813.99	8433.22	11790.22	28792.33	1.00E+00	NaN
rno-let-7c-1*	0.04	0.15	0.27	2.35	14.76	1.02E-07	NaN
rno-let-7d	218.80	2090.72	3021.18	4325.45	9045.13	3.86E-04	NaN
rno-let-7d*	0.15	6.63	11.02	17.19	51.65	1.71E-10	NaN
rno-let-7e	1.05	538.17	1224.57	1897.35	5184.52	4.55E-09	NaN
rno-let-7e*	0.04	0.10	0.15	0.25	1.05	1.00E+00	NaN
rno-let-7f	590.94	6021.56	9451.42	12156.47	22912.60	1.21E-07	NaN
rno-let-7f-1*	0.04	0.10	0.15	0.25	1.05	1.00E+00	NaN
rno-let-7f-2*	0.04	0.10	0.15	0.25	1.05	1.00E+00	NaN
rno-let-7i	432.57	1570.24	3054.18	4569.44	8432.51	1.98E-03	NaN
rno-let-7i*	0.04	0.10	0.15	0.25	9.35	5.45E-04	NaN
rno-miR-1	0.04	0.59	28.12	1022.22	158531.73	3.63E-04	NaN
rno-miR-1*	0.04	0.13	0.27	3.76	84.42	2.15E-07	NaN
rno-miR-100	0.32	147.83	330.78	609.36	2208.20	8.61E-08	NaN
rno-miR-100*	0.04	0.10	0.15	0.25	2.49	1.00E+00	NaN
rno-miR-101a	92.73	216.13	364.40	521.12	1626.91	1.00E+00	NaN
rno-miR-101a*	0.04	0.10	0.15	0.25	1.05	1.00E+00	NaN
rno-miR-101b	0.69	132.64	170.04	238.46	3695.66	1.03E-14	6
rno-miR-101b*	0.04	0.10	0.15	0.25	1.05	1.00E+00	NaN
rno-miR-103	0.66	592.23	856.15	1066.14	1943.70	2.45E-20	NaN
rno-miR-103-1*	0.04	0.10	0.15	0.25	1.05	1.00E+00	NaN
rno-miR-103-2*	0.04	0.10	0.15	0.25	1.05	1	NaN
rno-miR-105	0.04	0.10	0.15	0.25	6.54	1.05E-02	NaN
rno-miR-106b	1.05	189.31	292.88	433.68	2364.88	4.44E-07	NaN
rno-miR-106b*	0.04	0.11	0.17	0.30	28.20	4.10E-10	NaN
rno-miR-107	0.69	758.82	1049.45	1375.37	6350.00	2.12E-18	NaN
rno-miR-107*	0.04	0.10	0.15	0.25	1.05	1.00E+00	NaN
rno-miR-10a-3p	0.04	0.14	0.26	3.28	31.83	4.68E-09	NaN
rno-miR-10a-5p	0.08	107.73	813.28	1755.77	5844.59	1.32E-07	NaN
rno-miR-10b	0.04	67.78	405.06	1718.67	5033.55	1.89E-10	NaN
rno-miR-10b*	0.04	0.14	0.26	3.97	33.39	1.66E-08	NaN
rno-miR-1188-3p	0.05	6.21	14.55	25.71	119.77	1.56E-11	NaN
rno-miR-1188-5p	0.04	0.11	0.16	0.26	201.28	3.29E-15	8
rno-miR-1193-3p	0.04	0.10	0.15	0.25	1.05	1.00E+00	NaN
rno-miR-1193-5p	0.04	0.10	0.15	0.25	1.05	1	NaN
rno-miR-122	0.04	0.10	0.17	0.27	237948.69	1.83E-19	6
rno-miR-122*	0.04	0.10	0.15	0.25	6544.33	2.58E-20	6
rno-miR-1224	75.01	218.56	418.63	792.43	752897.90	4.84E-12	6
rno-miR-124	0.04	0.14	0.24	0.66	21435.00	1.80E-15	30
rno-miR-124*	0.04	0.13	0.20	0.32	35.59	7.23E-12	NaN
rno-miR-1249	0.04	0.12	0.25	14.28	413.90	5.82E-10	NaN
rno-miR-125a-3p	0.10	3.58	7.98	13.73	3683.65	6.08E-07	5
rno-miR-125a-5p	0.66	150.66	369.09	587.12	1078.25	1.32E-11	NaN
rno-miR-125b*	0.06	0.16	0.28	3.45	19.28	1.69E-08	NaN
rno-miR-125b-3p	0.04	0.10	0.15	0.25	1.05	1.00E+00	NaN
rno-miR-125b-5p	80.47	2268.48	4729.35	7736.27	33595.29	4.84E-06	NaN
rno-miR-126	95.16	533.36	1111.75	3040.94	10867.25	1.00E+00	NaN
rno-miR-126*	0.12	30.75	64.82	185.69	729.14	4.18E-10	NaN
rno-miR-127	0.06	0.33	35.30	270.18	2092.46	1.01E-07	NaN
rno-miR-127*	0.06	0.15	0.27	9.22	74.43	1.27E-11	NaN
rno-miR-128	0.66	61.76	110.78	493.04	10166.81	2.70E-06	12
rno-miR-128-1*	0.04	0.10	0.15	0.25	1.05	1.00E+00	NaN
rno-miR-128-2*	0.04	0.11	0.17	0.27	9.24	1.31E-09	NaN
rno-miR-129	0.04	0.14	0.23	0.64	794.15	1.55E-14	33
rno-miR-129-1*	0.06	7.83	13.37	31.37	2391.57	1.34E-05	NaN
rno-miR-129-2*	0.06	0.18	0.33	26.50	2838.37	5.43E-11	NaN
rno-miR-130a	1.05	422.65	754.01	1435.88	3143.45	1.50E-08	NaN
rno-miR-130a*	0.04	0.10	0.15	0.25	1.05	1.00E+00	NaN
rno-miR-130b	0.04	0.16	0.31	16.33	1214.23	2.34E-09	NaN
rno-miR-130b*	0.04	0.11	0.19	0.39	41.61	1.20E-10	NaN
rno-miR-132	0.06	0.17	0.27	8.36	1255.41	5.05E-12	30
rno-miR-132*	0.04	0.11	0.16	0.26	6.34	5.66E-06	NaN
rno-miR-133a	0.04	0.16	4.03	59.24	5011.82	3.26E-07	NaN
rno-miR-133a*	0.04	0.12	0.23	37.46	5935.05	1.58E-11	NaN
rno-miR-133b	0.18	33.24	188.85	2606.57	191928.27	0.023667643	NaN
rno-miR-133b*	0.04	0.10	0.15	0.25	1.05	1.00E+00	NaN
rno-miR-134	0.04	0.12	0.20	0.34	478.02	6.77E-13	31
rno-miR-134*	0.04	0.12	0.19	0.30	11.72	3.23E-11	NaN
rno-miR-135a	0.06	0.16	0.29	29.68	189.61	2.69E-11	NaN
rno-miR-135a*	0.04	0.12	0.18	0.29	31.84	6.93E-10	NaN
rno-miR-135b	0.06	0.15	0.27	21.71	277.53	3.49E-12	NaN
rno-miR-135b*	0.04	0.10	0.15	0.25	1.05	1	NaN
rno-miR-136	0.06	0.25	21.91	121.71	650.14	3.47E-08	NaN
rno-miR-136*	0.06	0.18	0.39	50.11	296.84	2.19E-10	NaN
rno-miR-137	0.06	0.14	0.23	0.61	627.29	4.13E-14	27
rno-miR-137*	0.04	0.12	0.17	0.27	8.60	4.73E-10	NaN
rno-miR-138	0.06	0.16	0.27	9.34	302.15	2.48E-12	27
rno-miR-138-1*	0.04	0.10	0.15	0.25	1.61	1	NaN
rno-miR-138-2*	0.04	0.13	0.20	0.33	18.75	9.68E-13	NaN
rno-miR-139-3p	0.08	10.92	19.65	28.99	5126.95	5.10E-10	8
rno-miR-139-5p	0.04	6.03	15.07	28.62	311.08	5.65E-09	NaN
rno-miR-140	0.66	204.35	269.11	353.18	10030.81	2.24E-14	8
rno-miR-140*	0.66	140.24	207.53	291.22	11001.85	2.46E-12	8
rno-miR-141	0.04	0.20	60.09	901.04	8798.65	8.83E-10	NaN
rno-miR-141*	0.04	0.10	0.16	0.25	5.62	2.71E-05	NaN
rno-miR-142-3p	0.69	138.15	336.86	746.27	53948.66	1.09E-05	15
rno-miR-142-5p	0.04	0.25	20.19	70.80	5412.80	1.47E-05	NaN
rno-miR-143	84.82	1078.62	3009.63	6557.55	83261.41	1	NaN
rno-miR-143*	0.04	0.13	0.22	0.64	97.33	2.40E-11	NaN
rno-miR-144	0.04	0.12	0.20	0.64	3290.75	1.84E-12	6
rno-miR-144*	0.04	0.12	0.17	0.31	186.03	9.82E-14	22
rno-miR-145	0.05	19.09	66.42	129.77	3396.35	7.12E-05	NaN
rno-miR-145*	0.04	0.11	0.17	0.27	42.00	5.64E-14	NaN
rno-miR-146a	30.98	227.01	423.47	791.65	3351.58	0.19575736	NaN
rno-miR-146a*	0.04	0.10	0.15	0.25	1.05	1	NaN
rno-miR-146b	0.49	62.61	93.85	132.40	537.37	4.71E-15	NaN
rno-miR-146b*	0.04	0.10	0.15	0.25	1.05	1	NaN
rno-miR-147	0.04	0.11	0.17	0.30	32.55	2.17E-12	NaN
rno-miR-148b-3p	0.66	122.74	161.12	212.00	465.38	1.27E-18	NaN
rno-miR-148b-5p	0.04	0.12	0.18	0.28	4.78	4.06E-06	NaN
rno-miR-150	0.66	105.59	216.92	582.12	14086.78	3.94E-04	9
rno-miR-150*	0.04	0.15	0.37	18.79	495.74	1.23E-07	NaN
rno-miR-151	0.66	427.76	565.99	801.16	1599.04	1.46E-18	NaN
rno-miR-151*	0.04	0.13	0.21	0.43	8.31	1.26E-07	NaN
rno-miR-152	2.87	68.16	135.46	224.96	1138.08	4.77E-02	NaN
rno-miR-152*	0.04	0.10	0.15	0.25	1.05	1.00E+00	NaN
rno-miR-153	0.06	0.16	0.27	10.36	242.15	3.54E-11	18
rno-miR-153*	0.04	0.10	0.15	0.25	1.05	1.00E+00	NaN
rno-miR-154	0.06	0.23	10.01	67.91	453.98	2.33E-09	NaN
rno-miR-154*	0.06	0.14	0.23	0.64	268.89	4.88E-13	NaN
rno-miR-15b	32.42	689.50	946.72	1473.16	13923.56	0.001740291	15
rno-miR-15b*	0.04	0.13	0.25	1.05	25.81	1.48E-07	NaN
rno-miR-16	175.81	2288.05	3249.48	4493.43	16073.99	7.06E-04	NaN
rno-miR-16*	0.04	0.10	0.15	0.25	1.05	1.00E+00	NaN
rno-miR-17-1-3p	0.04	0.12	0.20	0.39	31.27	1.28E-09	NaN
rno-miR-17-2-3p	0.04	0.10	0.15	0.25	1.05	1.00E+00	NaN
rno-miR-17-5p	0.66	43.54	75.97	130.46	1399.39	0.009362903	NaN
rno-miR-181a	102.77	301.09	544.85	938.27	17649.29	0.08351972	NaN
rno-miR-181a-1*	0.05	0.34	10.53	29.86	1086.48	2.93E-06	3
rno-miR-181a-2*	0.04	0.10	0.15	0.25	1.05	1.00E+00	NaN
rno-miR-181b	0.33	49.48	93.90	144.48	2639.77	1.88E-10	5
rno-miR-181b-1*	0.04	0.10	0.15	0.25	104.73	6.56E-15	3
rno-miR-181b-2*	0.04	0.10	0.15	0.25	1.05	1.00E+00	NaN
rno-miR-181c	0.33	51.45	64.06	93.04	419.34	1.17E-15	NaN
rno-miR-181c*	0.04	0.10	0.15	0.25	1.05	1	NaN
rno-miR-181d	0.28	29.56	40.93	54.59	264.35	7.58E-14	NaN
rno-miR-181d*	0.04	0.10	0.15	0.25	1.05	1.00E+00	NaN
rno-miR-182	0.04	0.22	7.37	28.07	405.23	2.45E-07	NaN
rno-miR-183	0.04	0.25	54.17	151.77	4643.37	1.23E-09	3
rno-miR-183*	0.04	0.11	0.20	0.33	50.48	6.73E-11	NaN
rno-miR-184	0.04	0.11	0.17	0.28	14536.44	3.84E-17	15
rno-miR-185	0.53	80.36	118.70	206.17	545.17	9.99E-11	NaN
rno-miR-185*	0.04	0.10	0.15	0.25	1.05	1.00E+00	NaN
rno-miR-186	74.90	119.75	153.84	208.21	385.44	1.00E+00	NaN
rno-miR-186*	0.04	0.10	0.15	0.25	1.05	1	NaN
rno-miR-187	0.04	0.10	0.15	0.25	1.05	1.00E+00	NaN
rno-miR-187*	0.04	0.10	0.15	0.25	32.01	3.91E-11	NaN
rno-miR-188	0.04	0.14	0.30	30.56	4285.67	1.18E-08	4
rno-miR-188*	0.04	0.11	0.17	0.28	95.27	1.09E-14	NaN
rno-miR-18a	0.05	0.27	6.44	28.29	665.88	1.01E-04	NaN
rno-miR-18a*	0.04	0.10	0.15	0.25	1.05	1.00E+00	NaN
rno-miR-190	0.05	0.13	0.22	0.56	29.40	5.69E-10	NaN
rno-miR-190*	0.04	0.11	0.17	0.28	11.36	1.44E-10	NaN
rno-miR-190b	0.04	0.10	0.15	0.25	1.05	1	NaN
rno-miR-190b*	0.04	0.10	0.15	0.25	1.05	1	NaN
rno-miR-191	0.04	0.10	0.17	0.27	12.93	3.73E-10	NaN
rno-miR-191*	0.22	8.84	13.77	20.62	72.24	2.32E-12	NaN
rno-miR-1912-3p	0.04	0.10	0.15	0.25	1.05	1	NaN
rno-miR-1912-5p	0.04	0.10	0.15	0.25	1.05	1.00E+00	NaN
rno-miR-192	0.22	16.65	31.09	53.94	38627.58	1.33E-10	33
rno-miR-192*	0.04	0.10	0.20	0.34	371.98	1.25E-14	33
rno-miR-193	0.08	51.76	111.89	206.11	8566.40	1.73E-04	NaN
rno-miR-193*	0.04	0.13	0.27	6.03	219.24	1.86E-08	NaN
rno-miR-194	0.08	0.35	9.57	17.46	39188.27	2.97E-07	13
rno-miR-194*	0.04	0.10	0.20	0.34	82.50	2.96E-13	NaN
rno-miR-1949	0.05	20.69	57.74	147.83	1029.60	1.72E-08	NaN
rno-miR-195	1.05	327.86	776.76	1827.52	6119.92	4.34E-05	NaN
rno-miR-195*	0.04	0.10	0.15	0.25	1.05	1.00E+00	NaN
rno-miR-196a	0.04	0.14	0.27	61.40	890.40	2.17E-11	NaN
rno-miR-196a*	0.04	0.10	0.15	0.25	1.05	1.00E+00	NaN
rno-miR-196b	0.04	0.14	0.30	131.12	1359.48	1.56E-10	NaN
rno-miR-196b*	0.04	0.10	0.15	0.25	1.05	1	NaN
rno-miR-196c	0.04	0.14	0.27	22.64	861.60	2.72E-09	NaN
rno-miR-196c*	0.04	0.11	0.19	0.31	405.68	8.47E-16	22
rno-miR-199a-3p	13.15	355.53	974.47	2118.58	9731.91	2.26E-02	NaN
rno-miR-199a-5p	0.04	85.46	215.36	436.09	3267.33	2.79E-10	NaN
rno-miR-19a	0.08	23.81	52.30	177.81	1593.88	1.24E-03	NaN
rno-miR-19a*	0.04	0.10	0.15	0.25	1.05	1.00E+00	NaN
rno-miR-19b	41.12	308.66	550.75	1267.64	7230.97	1	NaN
rno-miR-19b-1*	0.04	0.10	0.17	0.28	39.53	1.77E-13	NaN
rno-miR-19b-2*	0.04	0.10	0.15	0.25	1.05	1.00E+00	NaN
rno-miR-200a	0.04	0.21	219.88	2433.63	24103.62	3.47E-10	NaN
rno-miR-200a*	0.04	0.11	0.21	0.61	66.60	6.01E-12	NaN
rno-miR-200b	0.04	0.21	324.56	3718.40	40718.36	2.73E-10	NaN
rno-miR-200b*	0.04	0.15	0.35	47.52	408.48	3.62E-09	NaN
rno-miR-200c	0.04	0.17	4.11	238.36	1678.72	5.31E-10	NaN
rno-miR-200c*	0.04	0.10	0.15	0.25	1.05	1.00E+00	NaN
rno-miR-200c_v15.0	0.04	0.30	268.55	4255.80	22974.50	2.91E-08	NaN
rno-miR-201	0.04	0.11	0.17	0.28	435.09	3.43E-15	16
rno-miR-201*	0.04	0.10	0.16	0.27	34.83	6.54E-12	NaN
rno-miR-201_v15.0	0.04	0.11	0.17	0.28	288.72	2.79E-14	16
rno-miR-202	0.04	0.10	0.15	0.25	5.64	0.02992306	NaN
rno-miR-202*	0.04	0.13	0.26	7.74	316.00	4.91E-09	7
rno-miR-202_v15.0	0.04	0.10	0.15	0.25	71.39	1.97E-13	NaN
rno-miR-203	0.04	0.32	38.31	263.16	16980.79	5.63E-04	NaN
rno-miR-203*	0.04	0.10	0.15	0.25	1.05	1.00E+00	NaN
rno-miR-204	0.22	71.23	196.05	545.70	16085.60	5.68E-05	NaN
rno-miR-204*	0.04	0.13	0.21	0.34	41.27	2.17E-10	NaN
rno-miR-205	0.04	0.22	11.18	278.55	2563.11	1.05E-07	NaN
rno-miR-205_v15.0	0.04	0.23	21.09	401.87	6690.92	5.51E-07	NaN
rno-miR-206	0.04	0.12	0.23	8.49	28471.33	2.52E-13	19
rno-miR-206*	0.04	0.10	0.15	0.25	1.05	1.00E+00	NaN
rno-miR-207	0.04	0.10	0.15	0.25	24.12	8.93E-07	NaN
rno-miR-207_v15.0	0.04	0.10	0.15	0.25	16.73	1.10E-05	NaN
rno-miR-208	0.04	0.10	0.15	0.27	101.96	1.75E-16	12
rno-miR-208*	0.04	0.10	0.15	0.27	85.59	4.71E-16	NaN
rno-miR-208_v15.0	0.04	0.10	0.15	0.27	939.09	1.21E-18	12
rno-miR-208b-3p	0.04	0.10	0.15	0.25	53.09	6.48E-09	NaN
rno-miR-208b-5p	0.04	0.10	0.15	0.25	1.05	1	NaN
rno-miR-20a	0.66	152.50	272.69	524.16	4352.14	2.59E-06	NaN
rno-miR-20a*	0.04	0.16	0.33	4.81	64.68	1.10E-05	NaN
rno-miR-20b-3p	0.04	0.10	0.16	0.26	6.98	5.95E-07	NaN
rno-miR-20b-5p	0.66	81.30	145.67	272.59	2663.03	1.40E-04	NaN
rno-miR-20b-5p_v15.0	0.04	0.12	0.18	0.31	323.96	1.04E-13	24
rno-miR-21	373.94	2398.23	5423.12	13183.28	81002.91	1	NaN
rno-miR-21*	0.04	0.11	0.18	0.30	28.43	1.38E-10	NaN
rno-miR-210	0.23	31.30	60.01	124.81	449.33	5.74E-11	NaN
rno-miR-210*	0.04	0.10	0.15	0.25	1.05	1	NaN
rno-miR-211	0.07	0.17	0.29	3.64	3010.04	8.88E-10	3
rno-miR-211*	0.04	0.16	0.50	27.16	865.84	1.87E-07	NaN
rno-miR-212	0.06	0.25	8.51	21.78	243.00	5.49E-08	NaN
rno-miR-212*	0.04	0.11	0.17	0.27	7.29	2.12E-10	NaN
rno-miR-214	0.06	89.62	201.93	441.91	3191.01	1.83E-09	NaN
rno-miR-214*	0.04	0.10	0.15	0.25	1.05	1.00E+00	NaN
rno-miR-215	0.04	0.10	0.19	0.32	50159.24	2.32E-17	17
rno-miR-215_v15.0	0.04	0.10	0.19	0.33	83077.24	3.33E-17	17
rno-miR-216a	0.04	0.11	0.16	0.26	2713.17	2.88E-18	7
rno-miR-216a*	0.04	0.10	0.15	0.25	1.05	1	NaN
rno-miR-216b-3p	0.04	0.10	0.15	0.25	1.05	1	NaN
rno-miR-216b-5p	0.04	0.11	0.16	0.25	317.72	1.03E-16	6
rno-miR-217	0.04	0.11	0.16	0.25	1731.97	3.17E-18	5
rno-miR-217*	0.04	0.10	0.15	0.25	1.05	1.00E+00	NaN
rno-miR-217_v15.0	0.04	0.10	0.15	0.25	263.55	8.28E-17	3
rno-miR-218	0.09	34.91	72.44	239.79	1898.88	2.47E-10	NaN
rno-miR-218-1*	0.04	0.10	0.15	0.25	1.05	1	NaN
rno-miR-218-2*	0.04	0.10	0.15	0.25	1.05	1	NaN
rno-miR-219-1-3p	0.04	0.10	0.15	0.25	1.05	1	NaN
rno-miR-219-2-3p	0.04	0.13	0.20	0.34	1914.99	7.96E-17	27
rno-miR-219-5p	0.08	0.30	12.75	38.46	2717.50	1.69E-05	NaN
rno-miR-22	843.43	2160.93	4330.57	9292.37	32360.32	3.67E-03	NaN
rno-miR-22*	0.11	31.42	48.56	104.38	342.60	3.44E-12	NaN
rno-miR-220_v15.0	0.04	0.10	0.15	0.25	1.05	1	NaN
rno-miR-221	0.27	75.38	134.42	228.59	954.62	1.21E-11	NaN
rno-miR-221*	0.05	0.11	0.17	0.27	10.91	4.55E-10	NaN
rno-miR-222	0.05	0.63	19.31	48.50	282.61	2.98E-09	NaN
rno-miR-222*	0.04	0.10	0.15	0.25	1.05	1	NaN
rno-miR-223	23.93	187.66	299.18	467.34	5118.20	0.50673044	NaN
rno-miR-223*	0.04	0.10	0.15	0.25	1.05	1	NaN
rno-miR-224	0.04	0.11	0.17	0.27	8.53	1.57E-08	NaN
rno-miR-224*	0.04	0.10	0.16	0.26	7.23	2.50E-06	NaN
rno-miR-224_v15.0	0.04	0.15	2.06	50.02	316.76	2.65E-09	NaN
rno-miR-23a	518.56	2297.45	4144.09	10485.93	22126.41	2.84E-02	NaN
rno-miR-23a*	0.04	0.11	0.18	0.30	20.47	6.75E-10	NaN
rno-miR-23b	199.38	2860.93	4452.26	7512.34	31341.74	0.39951342	NaN
rno-miR-23b*	0.04	0.10	0.15	0.25	1.05	1.00E+00	NaN
rno-miR-24	338.79	1692.48	2632.61	5498.91	17408.16	1	NaN
rno-miR-24-1*	0.04	0.10	0.15	0.25	1.05	1	NaN
rno-miR-24-2*	0.22	11.68	23.87	60.70	222.18	4.96E-09	NaN
rno-miR-25	55.71	282.96	364.47	431.25	2319.58	1.76E-05	15
rno-miR-25*	0.04	0.10	0.15	0.25	1.05	1	NaN
rno-miR-26a	903.59	3240.30	5775.75	7505.45	15300.83	0.022537716	NaN
rno-miR-26a*	0.04	0.10	0.15	0.25	1.05	1	NaN
rno-miR-26b	257.77	1580.83	2622.57	3702.03	7388.00	1.27E-04	NaN
rno-miR-26b*	0.04	0.13	0.21	0.38	8.68	9.15E-08	NaN
rno-miR-27a	241.69	1051.70	2023.35	4124.49	9816.97	3.44E-02	NaN
rno-miR-27a*	0.04	0.10	0.15	0.25	1.05	1	NaN
rno-miR-27b	365.23	1234.89	1993.22	3007.14	10523.20	1.00E+00	NaN
rno-miR-27b*	0.04	0.10	0.15	0.25	1.05	1	NaN
rno-miR-28	0.66	81.12	152.59	231.23	773.62	5.23E-10	NaN
rno-miR-28*	0.04	0.11	0.17	0.27	12.45	1.41E-06	NaN
rno-miR-290	0.04	8.40	26.93	58.80	504.09	4.67E-11	NaN
rno-miR-290_v15.0	0.04	0.11	0.19	0.38	222.56	1.52E-12	34
rno-miR-291a-3p	0.04	0.10	0.15	0.25	1.05	1.00E+00	NaN
rno-miR-291a-5p	0.04	0.10	0.15	0.26	315.16	1.37E-15	9
rno-miR-291b	0.04	0.10	0.15	0.25	1.05	1	NaN
rno-miR-292-3p	0.04	0.10	0.15	0.25	1.05	1.00E+00	NaN
rno-miR-292-5p	0.04	0.10	0.17	0.28	124.35	1.43E-12	16
rno-miR-293	0.04	0.10	0.15	0.25	1.05	1	NaN
rno-miR-293*	0.04	0.10	0.15	0.25	1.05	1.00E+00	NaN
rno-miR-293_v15.0	0.04	0.10	0.15	0.25	1.05	1	NaN
rno-miR-294	0.04	0.10	0.15	0.25	7.42	2.86E-04	NaN
rno-miR-295	0.04	0.10	0.15	0.25	11.27	6.85E-06	NaN
rno-miR-295*	0.04	0.10	0.15	0.25	1.05	1.00E+00	NaN
rno-miR-295_v15.0	0.04	0.10	0.15	0.25	10.05	1.21E-05	NaN
rno-miR-296	0.04	0.10	0.15	0.25	1.05	1	NaN
rno-miR-296*	0.06	0.27	6.26	12.46	275.70	1.49E-06	NaN
rno-miR-2964	0.04	0.10	0.15	0.25	1.05	1	NaN
rno-miR-297	0.04	0.10	0.15	0.25	1.05	1	NaN
rno-miR-298	0.04	0.16	0.35	3.77	50.88	1.32E-06	NaN
rno-miR-298*	0.04	0.10	0.15	0.25	1.05	1	NaN
rno-miR-2985	14.52	36.11	57.58	92.83	334.21	1	NaN
rno-miR-299	0.06	0.22	8.07	35.75	580.29	5.12E-08	NaN
rno-miR-299*	0.04	0.11	0.16	0.25	3.07	9.76E-03	NaN
rno-miR-29a	1385.37	2855.73	4038.49	5158.04	10243.52	1.00E+00	NaN
rno-miR-29a*	0.06	3.80	6.14	8.75	18.16	3.41E-12	NaN
rno-miR-29b	564.99	1824.15	3005.00	4352.34	8235.58	0.032288026	NaN
rno-miR-29b-1*	0.04	0.10	0.15	0.25	1.21	1	NaN
rno-miR-29b-2*	0.04	0.12	0.18	0.28	2.23	3.68E-06	NaN
rno-miR-29c	279.41	1177.41	1809.01	2455.62	4203.73	0.003115777	NaN
rno-miR-29c*	0.18	33.60	69.00	111.90	213.56	5.27E-15	NaN
rno-miR-300-3p	0.06	0.23	9.86	98.29	382.83	4.95E-09	NaN
rno-miR-300-5p	0.06	0.14	0.21	0.38	24.50	2.47E-13	NaN
rno-miR-301a	0.64	76.14	106.91	138.00	502.36	1.37E-14	NaN
rno-miR-301a*	0.04	0.10	0.15	0.25	1.05	1.00E+00	NaN
rno-miR-301b	0.04	0.11	0.19	0.31	31.81	5.67E-11	NaN
rno-miR-301b*	0.04	0.10	0.15	0.25	1.05	1.00E+00	NaN
rno-miR-3065-3p	0.04	0.10	0.15	0.25	1.05	1.00E+00	NaN
rno-miR-3065-5p	0.04	0.10	0.15	0.25	1.05	1.00E+00	NaN
rno-miR-3074	0.04	0.10	0.15	0.25	1.05	1	NaN
rno-miR-3085	12.49	34.09	55.18	91.03	365.92	1	NaN
rno-miR-30a	241.54	533.22	992.92	2045.90	4579.74	2.48E-03	NaN
rno-miR-30a*	0.66	74.42	123.14	262.67	1047.27	6.97E-08	NaN
rno-miR-30b-3p	0.05	4.34	8.75	14.23	43.12	2.56E-11	NaN
rno-miR-30b-5p	117.83	636.72	948.75	1587.02	3418.97	0.28570926	NaN
rno-miR-30c	185.38	750.98	1126.84	1926.20	6611.22	8.33E-01	NaN
rno-miR-30c-1*	0.08	2.58	5.19	8.80	117.18	3.43E-04	NaN
rno-miR-30c-2*	0.11	13.16	22.62	50.80	109.04	4.15E-12	NaN
rno-miR-30d	205.44	412.59	599.76	913.21	2167.96	1.00E+00	NaN
rno-miR-30d*	0.04	0.13	0.24	1.65	12.27	1.46E-07	NaN
rno-miR-30e	195.39	376.31	612.28	1142.90	3760.12	1.01E-01	NaN
rno-miR-30e*	0.66	114.11	153.97	247.02	973.51	2.41E-13	NaN
rno-miR-31	0.07	0.28	20.69	99.53	1923.98	1.32E-06	NaN
rno-miR-31*	0.05	0.17	0.35	19.20	485.65	2.97E-08	NaN
rno-miR-3120	0.04	0.10	0.15	0.25	1.05	1.00E+00	NaN
rno-miR-32	0.04	0.16	0.35	7.33	65.21	4.94E-07	NaN
rno-miR-32*	0.04	0.10	0.16	0.25	111.41	8.65E-15	6
rno-miR-320	0.22	38.41	54.77	82.60	326.09	2.30E-13	NaN
rno-miR-320*	0.04	0.10	0.15	0.25	1.05	1.00E+00	NaN
rno-miR-322	0.66	96.72	204.54	559.02	4215.70	6.98E-03	NaN
rno-miR-322*	0.05	0.46	13.59	47.27	424.21	5.56E-08	NaN
rno-miR-323	0.06	0.14	0.23	0.61	145.83	4.96E-14	NaN
rno-miR-323*	0.04	0.10	0.15	0.25	1.05	1.00E+00	NaN
rno-miR-324-3p	97.09	153.53	190.21	228.06	567.56	0.9403239	NaN
rno-miR-324-5p	0.53	84.47	111.33	172.01	479.90	1.01E-15	NaN
rno-miR-325-3p	0.04	0.14	0.23	0.56	145.62	1.36E-14	33
rno-miR-325-5p	0.04	0.13	0.21	0.37	11.82	3.27E-11	NaN
rno-miR-326	0.13	15.71	32.45	58.49	253.35	5.62E-10	NaN
rno-miR-326*	0.04	0.10	0.15	0.25	62.22	2.56E-12	NaN
rno-miR-327	0.04	0.17	0.36	47.16	8657.61	5.35E-08	5
rno-miR-328a	0.66	58.14	95.08	157.00	571.77	3.52E-11	NaN
rno-miR-328a*	0.05	0.19	4.68	25.25	6184.15	7.92E-06	3
rno-miR-328b-3p	0.04	0.12	0.17	0.28	22.52	5.67E-10	NaN
rno-miR-329	0.06	0.23	10.54	154.81	569.60	2.72E-09	NaN
rno-miR-329*	0.04	0.10	0.15	0.25	1.05	1.00E+00	NaN
rno-miR-33	0.19	9.19	13.67	28.96	218.33	2.37E-07	NaN
rno-miR-33*	0.08	0.19	0.33	3.43	17.94	4.43E-08	NaN
rno-miR-330	0.04	0.12	0.19	0.30	4.33	1.75E-08	NaN
rno-miR-330*	0.07	0.15	0.25	2.24	104.32	2.27E-12	26
rno-miR-331	0.66	77.60	106.87	168.48	439.70	8.64E-15	NaN
rno-miR-331*	0.04	0.12	0.19	0.36	255.54	1.10E-13	29
rno-miR-335	0.09	7.13	58.89	148.06	2165.22	1.19E-08	NaN
rno-miR-336	0.04	0.10	0.15	0.25	1.05	1.00E+00	NaN
rno-miR-336*	0.04	0.10	0.15	0.25	1.05	1	NaN
rno-miR-337	0.06	0.19	0.50	22.14	193.43	2.32E-09	NaN
rno-miR-337*	0.06	0.15	0.27	10.81	101.89	7.08E-11	NaN
rno-miR-338	0.12	60.04	121.47	387.67	9238.49	8.28E-07	NaN
rno-miR-338*	0.04	0.13	0.21	0.38	31.56	1.34E-12	NaN
rno-miR-339-3p	0.04	0.12	0.21	0.38	9.32	1.05E-07	NaN
rno-miR-339-5p	0.04	0.10	0.15	0.25	2.24	1.00E+00	NaN
rno-miR-340-3p	0.13	9.41	18.28	30.28	125.05	1.27E-11	NaN
rno-miR-340-5p	0.13	0.28	16.95	41.46	104.19	7.08E-11	NaN
rno-miR-341	0.06	0.17	0.32	26.55	301.99	1.88E-10	NaN
rno-miR-342-3p	0.32	84.38	129.62	318.07	3113.84	1.90E-08	12
rno-miR-342-5p	0.07	0.20	0.53	5.99	107.70	3.94E-07	12
rno-miR-343	0.04	0.10	0.15	0.25	1.05	1.00E+00	NaN
rno-miR-344a	0.04	0.10	0.15	0.25	1.05	1.00E+00	NaN
rno-miR-344a-3p	0.04	0.14	0.22	0.49	172.03	2.61E-14	35
rno-miR-344a-5p	0.04	0.10	0.15	0.25	1.05	1.00E+00	NaN
rno-miR-344b-2-3p	0.04	0.12	0.17	0.28	18.80	2.40E-12	NaN
rno-miR-344b-5p	0.04	0.12	0.17	0.28	39.41	8.40E-14	NaN
rno-miR-345-3p	0.04	0.11	0.17	0.27	178.55	3.62E-13	16
rno-miR-345-5p	37.05	113.77	154.58	207.68	483.57	1.00E+00	NaN
rno-miR-346	0.04	0.13	0.19	0.31	8.74	4.32E-10	NaN
rno-miR-347	0.05	4.71	13.69	23.74	284.87	2.47E-08	NaN
rno-miR-349	0.04	0.10	0.15	0.25	1.05	1.00E+00	NaN
rno-miR-34a	30.20	215.22	441.72	915.14	5711.36	1	NaN
rno-miR-34a*	0.04	0.13	0.20	0.34	29.61	1.44E-12	NaN
rno-miR-34b	0.09	0.30	9.52	25.97	12120.03	2.57E-05	16
rno-miR-34b*	0.04	0.12	0.18	0.29	526.82	4.11E-15	16
rno-miR-34b_v15.0	0.09	0.27	9.26	36.52	18265.09	6.74E-06	15
rno-miR-34c	0.09	0.21	6.94	24.07	13443.18	7.45E-07	15
rno-miR-34c*	0.04	0.12	0.17	0.28	52.00	1.74E-12	NaN
rno-miR-350	0.08	0.25	3.36	7.25	14.86	2.88E-09	NaN
rno-miR-351	0.04	0.11	0.17	0.27	48.60	1.77E-11	NaN
rno-miR-351*	0.04	0.10	0.16	0.27	40.56	1.90E-13	NaN
rno-miR-352	0.66	108.90	154.39	230.93	662.42	8.53E-15	NaN
rno-miR-3541	0.04	0.10	0.15	0.25	78.79	6.81E-10	NaN
rno-miR-3542	0.04	0.10	0.15	0.25	1.05	1	NaN
rno-miR-3543	0.04	0.10	0.15	0.25	1.05	1.00E+00	NaN
rno-miR-3544	0.04	0.12	0.21	2.24	136.81	6.04E-10	NaN
rno-miR-3545-3p	0.04	0.10	0.18	0.30	124.42	3.07E-14	18
rno-miR-3545-5p	0.04	0.10	0.15	0.25	1.05	1.00E+00	NaN
rno-miR-3546	0.04	0.10	0.15	0.25	360.25	1.81E-17	5
rno-miR-3547	0.04	0.15	2.01	8.62	179.58	1.15E-07	NaN
rno-miR-3548	0.04	0.10	0.15	0.25	1.05	1	NaN
rno-miR-3549	0.04	0.13	0.29	11.77	293.89	2.55E-08	6
rno-miR-3550	0.04	0.10	0.15	0.25	24.91	7.21E-07	NaN
rno-miR-3551-3p	0.04	0.10	0.15	0.25	47.13	2.17E-11	NaN
rno-miR-3551-5p	0.04	0.10	0.15	0.25	1.05	1.00E+00	NaN
rno-miR-3552	0.04	0.10	0.15	0.25	1.05	1	NaN
rno-miR-3553	0.04	0.10	0.15	0.25	1.05	1	NaN
rno-miR-3554	0.04	0.10	0.15	0.25	35.86	4.18E-08	NaN
rno-miR-3555	0.04	0.10	0.15	0.25	1.05	1	NaN
rno-miR-3556a	0.04	0.10	0.15	0.25	1.05	1	NaN
rno-miR-3556b	0.04	0.10	0.15	0.25	1.05	1	NaN
rno-miR-3557-3p	0.04	0.10	0.17	0.27	115.08	3.94E-14	16
rno-miR-3557-5p	0.04	0.10	0.15	0.25	1.05	1	NaN
rno-miR-3558-3p	0.04	0.15	0.35	33.13	536.17	7.91E-09	NaN
rno-miR-3558-5p	0.04	0.10	0.15	0.25	1.05	1.00E+00	NaN
rno-miR-3559-3p	0.04	0.10	0.15	0.25	1.05	1	NaN
rno-miR-3559-5p	0.04	0.24	8.80	35.48	218.12	3.02E-07	NaN
rno-miR-3560	0.04	0.10	0.15	0.25	1.05	1	NaN
rno-miR-3561-3p	0.04	0.10	0.15	0.25	1.05	1	NaN
rno-miR-3561-5p	0.04	0.10	0.15	0.25	1.05	1	NaN
rno-miR-3562	0.04	0.11	0.19	0.34	171.89	1.37E-12	33
rno-miR-3563-3p	0.04	0.11	0.17	0.28	31.40	8.41E-14	NaN
rno-miR-3563-5p	0.04	0.11	0.17	0.27	4.68	1.68E-05	NaN
rno-miR-3564	0.04	0.11	0.21	0.49	694.83	1.89E-13	37
rno-miR-3565	0.04	0.10	0.15	0.25	1.05	1.00E+00	NaN
rno-miR-3566	0.04	0.10	0.15	0.25	1.05	1	NaN
rno-miR-3567	0.04	0.10	0.15	0.25	1.05	1	NaN
rno-miR-3568	0.04	0.10	0.15	0.25	5.97	2.05E-02	NaN
rno-miR-3569	0.04	0.10	0.15	0.25	1.05	1	NaN
rno-miR-3570	0.04	0.10	0.15	0.25	1.05	1	NaN
rno-miR-3571	0.04	0.10	0.15	0.25	1.05	1.00E+00	NaN
rno-miR-3572	0.04	0.10	0.15	0.25	7.48	0.003819167	NaN
rno-miR-3573-3p	6.44	16.68	26.78	42.66	262.95	1	NaN
rno-miR-3573-5p	0.04	0.10	0.15	0.25	20.68	2.58E-06	NaN
rno-miR-3574	0.04	0.10	0.15	0.25	1.05	1.00E+00	NaN
rno-miR-3575	0.04	0.10	0.15	0.25	1.05	1	NaN
rno-miR-3576	0.04	0.10	0.15	0.25	1.05	1	NaN
rno-miR-3577	0.04	0.10	0.15	0.25	1.05	1	NaN
rno-miR-3578	0.04	0.10	0.15	0.25	1.05	1	NaN
rno-miR-3579	0.04	0.10	0.15	0.25	1.05	1	NaN
rno-miR-3580-3p	0.04	0.12	0.19	0.34	1014.44	1.90E-14	28
rno-miR-3580-5p	0.04	0.10	0.15	0.25	1.05	1.00E+00	NaN
rno-miR-3581	0.04	0.10	0.15	0.25	1.05	1	NaN
rno-miR-3582	0.04	0.10	0.17	0.28	107.42	4.72E-14	16
rno-miR-3583-3p	0.04	0.10	0.15	0.25	1.05	1	NaN
rno-miR-3583-5p	0.04	0.10	0.15	0.25	1.05	1	NaN
rno-miR-3584-3p	0.04	0.10	0.15	0.25	25.37	6.39E-07	NaN
rno-miR-3584-5p	0.05	18.94	45.51	170.77	167704.17	6.16E-04	17
rno-miR-3585-3p	0.04	0.10	0.15	0.25	1.05	1	NaN
rno-miR-3585-5p	0.04	0.11	0.18	0.38	498.76	1.18E-12	31
rno-miR-3586-3p	0.04	0.10	0.15	0.25	1.05	1	NaN
rno-miR-3586-5p	0.04	0.10	0.15	0.25	1.05	1.00E+00	NaN
rno-miR-3587	0.04	0.10	0.15	0.25	1.05	1.00E+00	NaN
rno-miR-3588	0.08	34.71	94.50	161.39	1283.51	2.39E-12	NaN
rno-miR-3589	0.04	0.10	0.15	0.25	1.05	1.00E+00	NaN
rno-miR-3590-3p	0.04	0.10	0.15	0.25	1.05	1.00E+00	NaN
rno-miR-3590-5p	0.04	0.10	0.15	0.25	1.05	1	NaN
rno-miR-3591	0.04	0.10	0.15	0.25	1.05	1.00E+00	NaN
rno-miR-3592	0.04	0.10	0.15	0.25	1.05	1	NaN
rno-miR-3593-3p	0.04	0.13	0.31	29.77	809.20	1.68E-09	NaN
rno-miR-3593-5p	0.04	0.11	0.17	0.30	132.07	2.72E-14	20
rno-miR-3594-3p	0.04	0.10	0.15	0.25	1.05	1.00E+00	NaN
rno-miR-3594-5p	0.04	0.10	0.16	0.26	37.19	1.53E-11	NaN
rno-miR-3595	0.04	0.10	0.15	0.25	1.05	1	NaN
rno-miR-3596a	0.04	0.10	0.15	0.25	1.05	1.00E+00	NaN
rno-miR-3596b	0.04	0.10	0.15	0.25	1.05	1.00E+00	NaN
rno-miR-3596c	0.04	0.10	0.15	0.25	1.05	1	NaN
rno-miR-3596d	0.04	0.10	0.15	0.25	1.05	1.00E+00	NaN
rno-miR-3597-3p	0.04	0.10	0.15	0.25	1.05	1	NaN
rno-miR-3597-5p	0.04	0.10	0.15	0.25	1.05	1.00E+00	NaN
rno-miR-361	0.66	92.90	129.77	160.59	517.80	1.99E-14	NaN
rno-miR-361*	0.04	0.12	0.17	0.28	7.86	2.17E-09	NaN
rno-miR-362	0.04	0.27	4.90	12.77	46.66	4.36E-07	NaN
rno-miR-362*	0.28	25.38	45.62	75.92	180.15	8.85E-11	NaN
rno-miR-363	0.04	0.13	0.25	5.11	373.45	3.50E-10	18
rno-miR-363*	0.04	0.10	0.15	0.25	1.05	1.00E+00	NaN
rno-miR-363_v15.0	0.04	0.15	0.30	13.32	759.81	1.92E-08	19
rno-miR-365	45.52	142.17	393.00	631.97	7466.02	1.00E+00	NaN
rno-miR-365*	0.04	0.10	0.15	0.25	1.05	1	NaN
rno-miR-369-3p	0.06	0.13	0.21	0.34	30.60	3.15E-13	NaN
rno-miR-369-5p	0.06	0.18	0.39	46.37	238.33	2.53E-10	NaN
rno-miR-370	0.06	0.16	0.30	11.38	6400.64	2.71E-09	3
rno-miR-370*	0.04	0.10	0.15	0.25	1.05	1.00E+00	NaN
rno-miR-370_v15.0	0.04	0.11	0.16	0.26	425.72	3.95E-16	3
rno-miR-374	0.12	3.35	6.66	10.15	42.00	2.00E-09	NaN
rno-miR-374*	0.04	0.10	0.15	0.25	1.05	1.00E+00	NaN
rno-miR-375	0.04	0.17	0.57	435.36	5547.82	4.68E-10	NaN
rno-miR-375*	0.04	0.10	0.15	0.25	1.05	1.00E+00	NaN
rno-miR-376a	0.06	0.21	0.54	37.42	535.99	5.48E-09	NaN
rno-miR-376a*	0.06	0.13	0.21	0.38	14.63	1.33E-11	NaN
rno-miR-376b-3p	0.06	0.15	0.25	12.15	338.83	1.56E-12	NaN
rno-miR-376b-5p	0.06	0.17	0.32	9.17	65.89	2.31E-09	NaN
rno-miR-376c	0.06	0.19	0.39	19.64	286.94	8.13E-09	NaN
rno-miR-376c*	0.04	0.10	0.15	0.25	1.05	1.00E+00	NaN
rno-miR-377	0.05	0.11	0.17	0.27	14.26	9.50E-12	NaN
rno-miR-377*	0.04	0.10	0.15	0.25	1.05	1.00E+00	NaN
rno-miR-377_v15.0	0.06	0.13	0.21	0.39	62.78	2.41E-12	NaN
rno-miR-378	28.04	157.43	388.76	1103.04	12683.83	1.00E+00	NaN
rno-miR-378*	0.06	22.50	60.38	211.05	1932.92	3.46E-05	NaN
rno-miR-379	0.06	0.24	11.27	103.85	449.41	2.43E-09	NaN
rno-miR-379*	0.06	0.16	0.28	6.91	37.43	1.11E-10	NaN
rno-miR-380	0.04	0.12	0.17	0.27	66.00	6.69E-09	NaN
rno-miR-380*	0.06	0.14	0.23	0.53	139.53	4.20E-14	37
rno-miR-381	0.06	0.18	0.35	50.14	385.12	4.22E-11	NaN
rno-miR-381*	0.06	0.13	0.21	0.39	25.86	9.19E-11	NaN
rno-miR-381_v15.0	0.04	0.11	0.16	0.25	1.94	1.12E-01	NaN
rno-miR-382	0.06	0.20	0.57	29.66	229.16	2.96E-09	NaN
rno-miR-382*	0.06	0.14	0.23	0.54	79.77	1.12E-13	NaN
rno-miR-383	0.04	0.14	0.22	0.39	186.17	1.51E-14	32
rno-miR-383*	0.04	0.10	0.15	0.25	1.05	1.00E+00	NaN
rno-miR-383_v15.0	0.04	0.13	0.21	0.37	128.08	1.93E-14	31
rno-miR-384-3p	0.04	0.13	0.22	0.39	30.03	1.75E-12	NaN
rno-miR-384-5p	0.06	0.15	0.25	8.71	703.42	4.20E-14	33
rno-miR-409-3p	11.14	32.35	48.29	76.19	283.30	1.00E+00	NaN
rno-miR-409-5p	0.06	0.15	0.25	11.91	111.01	1.74E-12	NaN
rno-miR-409-5p_v15.0	0.06	0.14	0.23	0.66	43.44	5.91E-12	NaN
rno-miR-410	0.06	0.17	0.30	47.07	403.41	3.88E-11	NaN
rno-miR-410*	0.04	0.10	0.15	0.25	1.05	1	NaN
rno-miR-411	0.06	0.18	0.37	40.19	150.32	1.46E-10	NaN
rno-miR-411*	0.06	0.22	7.05	106.44	329.22	3.37E-10	NaN
rno-miR-412	0.04	0.10	0.15	0.25	1.05	1	NaN
rno-miR-412*	0.04	0.11	0.16	0.26	3.90	2.99E-06	NaN
rno-miR-421	0.04	0.10	0.15	0.25	1.05	1.00E+00	NaN
rno-miR-421*	0.10	0.22	0.54	9.87	29.73	2.98E-10	NaN
rno-miR-423	0.04	0.11	0.16	0.26	40.50	1.18E-10	NaN
rno-miR-423*	0.38	17.65	27.47	39.71	298.32	1.28E-08	NaN
rno-miR-425	0.64	43.33	68.48	108.07	440.43	8.54E-11	NaN
rno-miR-425*	0.04	0.10	0.15	0.25	18.44	3.79E-06	NaN
rno-miR-429	0.04	0.21	123.53	1486.43	10686.49	1.94E-10	NaN
rno-miR-431	0.06	0.15	0.27	11.74	300.11	1.54E-10	10
rno-miR-433	0.06	0.14	0.25	8.71	249.28	2.37E-13	32
rno-miR-433*	0.06	0.14	0.22	0.49	44.11	1.90E-13	NaN
rno-miR-434	0.06	0.27	35.25	337.29	1430.38	1.32E-08	NaN
rno-miR-434*	0.06	0.15	0.27	12.25	93.55	1.25E-11	NaN
rno-miR-448	0.04	0.12	0.17	0.28	15.59	3.59E-10	NaN
rno-miR-448*	0.04	0.12	0.17	0.27	7.93	2.58E-10	NaN
rno-miR-448_v15.0	0.04	0.10	0.15	0.25	7.45	0.001404246	NaN
rno-miR-449a	0.04	0.13	0.24	0.88	5573.39	3.00E-12	15
rno-miR-449a*	0.04	0.10	0.15	0.25	1.05	1	NaN
rno-miR-449c-3p	0.04	0.10	0.15	0.25	1.05	1.00E+00	NaN
rno-miR-449c-5p	0.04	0.11	0.17	0.27	4.92	5.17E-05	NaN
rno-miR-450a	0.12	14.61	38.98	96.74	1039.39	1.31E-09	NaN
rno-miR-450a*	0.04	0.10	0.15	0.25	10.05	5.28E-07	NaN
rno-miR-450a_v15.0	0.04	0.17	0.67	21.04	255.90	8.70E-08	NaN
rno-miR-451	0.04	8.96	65.30	276.08	36495.07	7.24E-02	NaN
rno-miR-451*	0.04	0.10	0.15	0.25	1.05	1	NaN
rno-miR-455	0.04	0.12	0.20	0.34	92.19	9.07E-13	NaN
rno-miR-455*	0.12	11.45	26.24	57.54	1000.44	1.26E-09	NaN
rno-miR-463	0.04	0.10	0.16	0.27	785.95	9.62E-18	7
rno-miR-463*	0.04	0.10	0.15	0.25	12.26	9.11E-08	NaN
rno-miR-465	0.04	0.10	0.15	0.25	5.71	0.028226383	NaN
rno-miR-465*	0.04	0.10	0.15	0.26	25.14	6.67E-11	NaN
rno-miR-466b	0.05	6.11	13.57	23.81	2351.78	8.53E-07	9
rno-miR-466b-1*	7.99	35.75	63.40	118.92	440.03	1.00E+00	NaN
rno-miR-466b-2*	0.04	0.21	3.86	17.00	199.69	5.04E-07	NaN
rno-miR-466c	0.04	0.10	0.15	0.25	1.05	1.00E+00	NaN
rno-miR-466c*	0.04	0.10	0.16	0.26	17.99	8.57E-11	NaN
rno-miR-466d	0.04	0.10	0.15	0.25	1.05	1.00E+00	NaN
rno-miR-471	0.04	0.10	0.16	0.27	761.81	2.91E-17	8
rno-miR-471*	0.04	0.10	0.16	0.27	661.55	2.90E-17	7
rno-miR-483	0.04	3.19	10.82	19.88	152.51	1.43E-09	NaN
rno-miR-483*	0.05	0.20	4.16	26.93	2233.57	1.32E-04	NaN
rno-miR-484	0.10	9.28	14.34	17.90	86.14	9.32E-14	NaN
rno-miR-485	0.06	0.14	0.23	0.61	47.93	3.87E-13	NaN
rno-miR-485*	0.04	0.10	0.15	0.25	1.05	1	NaN
rno-miR-487b	0.06	0.19	0.50	47.18	393.01	1.04E-09	NaN
rno-miR-487b*	0.04	0.10	0.15	0.25	1.05	1.00E+00	NaN
rno-miR-487b_v15.0	0.06	0.15	0.25	5.11	65.72	2.52E-11	NaN
rno-miR-488	0.04	0.13	0.21	0.37	19.70	1.75E-12	NaN
rno-miR-488*	0.04	0.10	0.15	0.25	1.05	1	NaN
rno-miR-489	0.04	0.11	0.16	0.27	5.70	3.44E-08	NaN
rno-miR-489*	0.04	0.10	0.15	0.25	1.05	1.00E+00	NaN
rno-miR-490	0.04	0.13	0.22	1.41	162.94	3.22E-12	21
rno-miR-490*	0.04	0.14	0.23	5.55	85.26	1.42E-11	NaN
rno-miR-493	0.04	0.11	0.16	0.26	362.36	3.50E-16	7
rno-miR-493*	0.04	0.11	0.16	0.26	7.96	3.40E-08	NaN
rno-miR-494	17.53	83.14	134.14	246.16	60962.48	1.38E-10	11
rno-miR-494*	0.04	0.10	0.15	0.25	1.05	1.00E+00	NaN
rno-miR-494_v15.0	22.42	117.53	205.23	352.73	170953.80	3.27E-11	10
rno-miR-495	0.06	0.17	0.32	38.19	304.66	9.24E-11	NaN
rno-miR-496	0.06	0.13	0.21	0.36	15.30	1.43E-12	NaN
rno-miR-496*	0.04	0.10	0.15	0.25	1.05	1.00E+00	NaN
rno-miR-496_v15.0	0.06	0.14	0.23	0.64	52.92	2.24E-13	NaN
rno-miR-497	69.62	295.98	658.38	1389.89	4721.54	4.95E-01	NaN
rno-miR-497*	0.04	0.10	0.15	0.25	1.05	1	NaN
rno-miR-499	0.11	8.57	17.70	43.21	2232.83	4.08E-05	18
rno-miR-499*	0.04	0.10	0.15	0.25	1.05	1	NaN
rno-miR-500	30.07	114.63	159.30	231.00	1074.50	2.69E-01	NaN
rno-miR-500*	0.04	0.10	0.15	0.25	1.05	1.00E+00	NaN
rno-miR-501	0.04	0.10	0.15	0.25	1.05	1.00E+00	NaN
rno-miR-501*	0.04	0.10	0.15	0.25	37.84	3.05E-08	NaN
rno-miR-501_v15.0	0.04	0.10	0.15	0.25	1.05	1.00E+00	NaN
rno-miR-503	0.04	0.15	0.45	16.94	71.56	1.01E-08	NaN
rno-miR-503*	0.04	0.10	0.15	0.25	1.05	1.00E+00	NaN
rno-miR-504	0.04	0.10	0.15	0.25	1.05	1.00E+00	NaN
rno-miR-505	0.24	8.82	12.64	18.64	36.73	3.38E-14	NaN
rno-miR-505*	0.07	0.19	0.33	6.97	13.07	1.27E-10	NaN
rno-miR-511	0.04	0.10	0.15	0.25	1.05	1	NaN
rno-miR-511*	0.04	0.27	18.10	36.97	164.31	1.17E-09	NaN
rno-miR-511_v15.0	0.04	0.10	0.15	0.25	1.05	1.00E+00	NaN
rno-miR-532-3p	0.30	11.76	18.01	25.98	104.57	4.05E-11	NaN
rno-miR-532-5p	0.38	24.22	46.55	63.28	220.53	8.85E-09	NaN
rno-miR-539	0.06	0.17	0.35	13.34	68.50	2.92E-09	NaN
rno-miR-539*	0.04	0.10	0.15	0.25	1.05	1	NaN
rno-miR-540	0.04	0.10	0.15	0.25	4.26	1.09E-03	NaN
rno-miR-540*	0.04	0.10	0.15	0.25	1.15	1.00E+00	NaN
rno-miR-540_v15.0	0.04	0.11	0.17	0.27	6.52	1.18E-06	NaN
rno-miR-541	0.06	0.15	0.25	9.14	67.13	6.64E-12	NaN
rno-miR-541*	0.04	0.11	0.17	0.27	15.19	1.94E-11	NaN
rno-miR-542-3p	0.05	0.32	9.15	29.89	439.16	3.04E-07	3
rno-miR-542-5p	0.05	0.27	10.09	42.04	332.95	3.51E-08	NaN
rno-miR-543	0.04	0.10	0.15	0.25	1.05	1	NaN
rno-miR-543*	0.06	0.15	0.25	6.97	123.49	6.60E-13	NaN
rno-miR-543_v15.0	0.06	0.13	0.21	0.34	6.68	2.43E-09	NaN
rno-miR-544	0.04	0.10	0.15	0.25	1.05	1.00E+00	NaN
rno-miR-544*	0.04	0.10	0.15	0.25	1.05	1	NaN
rno-miR-544_v15.0	0.04	0.10	0.15	0.25	1.05	1	NaN
rno-miR-547	0.04	0.11	0.18	0.36	590.02	2.20E-13	6
rno-miR-547*	0.04	0.10	0.15	0.26	53.27	2.66E-13	NaN
rno-miR-547_v15.0	0.04	0.10	0.16	0.27	83.96	3.92E-14	NaN
rno-miR-551b	0.04	0.15	0.24	0.72	1606.61	7.81E-14	24
rno-miR-551b*	0.04	0.10	0.15	0.25	1.05	1.00E+00	NaN
rno-miR-568	0.04	0.10	0.15	0.25	1.05	1.00E+00	NaN
rno-miR-582	0.07	10.02	29.92	62.51	292.17	6.17E-11	NaN
rno-miR-582*	0.06	0.18	2.43	9.86	76.63	1.60E-07	NaN
rno-miR-592	0.04	0.13	0.20	0.32	28.42	1.44E-12	NaN
rno-miR-598-3p	0.09	0.23	0.75	31.30	549.92	2.96E-08	NaN
rno-miR-598-5p	0.04	0.10	0.15	0.25	1.05	1.00E+00	NaN
rno-miR-615	0.04	0.10	0.15	0.25	1.05	1.00E+00	NaN
rno-miR-628	0.04	0.10	0.15	0.25	1.05	1	NaN
rno-miR-632	0.04	0.10	0.15	0.25	1.05	1.00E+00	NaN
rno-miR-652	120.14	223.45	322.41	550.64	8315.28	6.36E-07	3
rno-miR-652*	7.64	43.83	77.36	135.38	8521.49	5.71E-05	3
rno-miR-653	0.04	0.11	0.17	0.27	12.47	1.83E-11	NaN
rno-miR-653*	0.04	0.10	0.15	0.25	1.05	1.00E+00	NaN
rno-miR-653_v15.0	0.04	0.10	0.15	0.25	1.05	1.00E+00	NaN
rno-miR-664	10.92	29.60	38.83	53.96	202.78	1	NaN
rno-miR-664-1*	0.04	0.10	0.17	0.28	110.07	1.77E-14	17
rno-miR-664-2*	0.04	0.12	0.18	0.29	15.86	4.76E-10	NaN
rno-miR-665	0.06	0.12	0.18	0.29	11.78	8.61E-11	NaN
rno-miR-665_v15.0	0.04	0.11	0.17	0.27	17.09	4.11E-08	NaN
rno-miR-666	0.04	0.10	0.15	0.25	1619.00	1.03E-18	3
rno-miR-666*	0.04	0.10	0.15	0.25	1.05	1	NaN
rno-miR-666_v15.0	0.04	0.10	0.15	0.25	219.96	6.46E-16	4
rno-miR-667	0.06	0.17	0.28	9.34	51.51	8.64E-11	NaN
rno-miR-667*	0.04	0.10	0.16	0.26	56.34	3.07E-12	NaN
rno-miR-667_v15.0	0.04	0.13	0.20	0.32	5.22	9.24E-10	NaN
rno-miR-668	0.04	0.10	0.15	0.25	12.40	9.27E-05	NaN
rno-miR-671	0.04	0.10	0.15	0.25	8.22	1.90E-03	NaN
rno-miR-672	0.09	0.21	8.84	64.83	947.30	1.03E-08	NaN
rno-miR-672*	0.04	0.10	0.15	0.25	1.05	1.00E+00	NaN
rno-miR-673	0.04	0.10	0.15	0.25	1.05	1.00E+00	NaN
rno-miR-673*	0.04	0.10	0.15	0.25	1.05	1.00E+00	NaN
rno-miR-674-3p	0.08	7.30	11.78	18.83	33.72	3.66E-13	NaN
rno-miR-674-5p	0.05	0.17	0.28	2.79	8.47	1.32E-08	NaN
rno-miR-675	0.04	0.10	0.15	0.25	1.05	1.00E+00	NaN
rno-miR-675*	0.04	0.11	0.17	0.31	330.99	5.69E-15	25
rno-miR-678	0.04	0.10	0.17	0.27	152.95	5.19E-15	11
rno-miR-702-3p	13.14	31.60	52.15	82.56	282.00	1	NaN
rno-miR-702-5p	0.04	0.10	0.15	0.25	1.05	1.00E+00	NaN
rno-miR-708	0.04	0.10	0.15	0.25	3.01	1.00E+00	NaN
rno-miR-708*	0.04	0.10	0.15	0.25	1.05	1	NaN
rno-miR-711	0.04	0.10	0.15	0.25	9.44	4.45E-05	NaN
rno-miR-741-3p	0.04	0.10	0.16	0.27	3094.37	1.18E-18	9
rno-miR-741-5p	0.04	0.10	0.15	0.25	1.05	1.00E+00	NaN
rno-miR-742	0.04	0.10	0.16	0.27	795.81	9.51E-18	11
rno-miR-742*	0.04	0.10	0.16	0.27	332.55	5.09E-17	10
rno-miR-743a	0.04	0.10	0.16	0.27	454.12	2.23E-17	11
rno-miR-743a*	0.04	0.10	0.16	0.27	90.13	6.48E-15	NaN
rno-miR-743b	0.04	0.10	0.16	0.27	1633.21	1.48E-18	11
rno-miR-743b*	0.04	0.10	0.16	0.27	165.57	2.79E-16	9
rno-miR-758	0.06	0.14	0.22	0.39	105.29	3.60E-14	31
rno-miR-758*	0.04	0.10	0.15	0.25	1.05	1.00E+00	NaN
rno-miR-759	0.04	0.10	0.15	0.25	1.05	1.00E+00	NaN
rno-miR-760-3p	0.05	0.14	0.24	1.71	98.91	1.45E-09	NaN
rno-miR-760-5p	0.04	0.10	0.16	0.26	191.93	8.64E-15	8
rno-miR-761	0.04	0.10	0.15	0.25	1.05	1	NaN
rno-miR-764	0.04	0.10	0.15	0.25	1.05	1.00E+00	NaN
rno-miR-764*	0.04	0.11	0.17	0.27	4.38	1.35E-06	NaN
rno-miR-764_v15.0	0.04	0.12	0.18	0.28	7.53	1.30E-09	NaN
rno-miR-770	0.04	0.10	0.15	0.25	1.05	1	NaN
rno-miR-770*	0.06	0.14	0.21	0.41	439.91	3.73E-13	36
rno-miR-7a	17.69	40.39	73.38	254.21	21015.92	2.57E-07	3
rno-miR-7a-1*	0.22	9.06	13.98	25.05	84.92	1.17E-08	NaN
rno-miR-7a-2*	0.04	0.12	0.17	0.28	177.53	4.30E-15	12
rno-miR-7b	0.04	0.19	0.41	14.70	5325.66	6.82E-08	6
rno-miR-802	0.04	0.10	0.16	0.27	49.84	2.36E-15	NaN
rno-miR-802*	0.04	0.10	0.15	0.25	1.05	1.00E+00	NaN
rno-miR-802_v15.0	0.04	0.11	0.17	0.28	298.16	1.17E-16	18
rno-miR-871	0.04	0.10	0.15	0.25	1.05	1.00E+00	NaN
rno-miR-871*	0.04	0.10	0.16	0.27	603.88	2.07E-17	6
rno-miR-871_v15.0	0.04	0.10	0.16	0.27	22.48	3.02E-12	NaN
rno-miR-872	0.64	47.14	63.84	83.29	253.45	9.13E-16	NaN
rno-miR-872*	0.05	0.14	0.24	0.66	10.50	1.70E-07	NaN
rno-miR-873	0.04	0.13	0.21	0.39	45.10	7.13E-12	NaN
rno-miR-873*	0.04	0.10	0.15	0.25	1.05	1	NaN
rno-miR-874	0.04	0.21	1.34	4.93	832.60	2.62E-07	4
rno-miR-874*	0.04	0.10	0.15	0.25	1.05	1.00E+00	NaN
rno-miR-875	0.04	0.10	0.15	0.25	1.05	1	NaN
rno-miR-876	0.04	0.10	0.15	0.25	1.05	1.00E+00	NaN
rno-miR-877	0.04	0.11	0.19	0.34	187.38	1.22E-12	30
rno-miR-878	0.04	0.10	0.15	0.25	11.70	1.63E-07	NaN
rno-miR-879	0.04	0.10	0.15	0.25	1.05	1.00E+00	NaN
rno-miR-879*	0.04	0.10	0.15	0.25	1.05	1.00E+00	NaN
rno-miR-880	0.04	0.10	0.16	0.27	520.57	1.34E-17	7
rno-miR-880*	0.04	0.10	0.15	0.25	1.05	1	NaN
rno-miR-881	0.04	0.10	0.15	0.25	19.00	6.71E-10	NaN
rno-miR-881*	0.04	0.10	0.16	0.27	59.57	2.69E-14	NaN
rno-miR-881_v15.0	0.04	0.10	0.16	0.27	480.61	1.21E-17	6
rno-miR-883	0.04	0.10	0.16	0.27	1019.83	6.28E-18	11
rno-miR-883*	0.04	0.10	0.16	0.27	344.36	4.11E-17	12
rno-miR-9	0.06	0.19	0.67	45.69	5173.33	1.90E-08	NaN
rno-miR-9*	0.06	0.18	0.41	55.45	8290.03	5.73E-10	NaN
rno-miR-92a	30.51	270.04	447.36	805.31	6072.39	1	NaN
rno-miR-92a-1*	0.04	0.10	0.15	0.25	1.05	1	NaN
rno-miR-92a-2*	0.04	0.10	0.16	0.27	19.81	9.73E-12	NaN
rno-miR-92b	0.04	0.15	0.28	3.70	62.60	3.00E-07	NaN
rno-miR-92b*	0.04	0.10	0.15	0.25	1.05	1	NaN
rno-miR-93	0.66	76.99	122.35	185.60	1312.71	4.93E-11	6
rno-miR-93*	0.04	0.10	0.15	0.25	1.05	1	NaN
rno-miR-935	0.04	0.10	0.15	0.25	1.05	1	NaN
rno-miR-96	0.04	0.28	111.69	447.51	9114.44	9.25E-09	NaN
rno-miR-96*	0.04	0.10	0.16	0.25	7.75	3.77E-07	NaN
rno-miR-98	1.05	251.33	342.31	409.05	750.08	3.82E-17	NaN
rno-miR-98*	0.04	0.10	0.15	0.25	1.05	1	NaN
rno-miR-99a	15.96	919.18	1485.49	2515.58	5746.90	1.94E-10	NaN
rno-miR-99a*	0.07	7.03	16.18	27.46	52.84	1.18E-12	NaN
rno-miR-99b	0.14	54.23	118.53	208.20	823.34	4.90E-11	NaN
rno-miR-99b*	0.05	0.17	0.27	2.31	181.48	1.44E-08	3
rno-miR-218a	NaN	NaN	NaN	NaN	NaN	NaN	NaN
rno-miR-218a-1*	NaN	NaN	NaN	NaN	NaN	NaN	NaN
rno-miR-218a-2*	NaN	NaN	NaN	NaN	NaN	NaN	NaN
rno-miR-218b	NaN	NaN	NaN	NaN	NaN	NaN	NaN

**Table 5:** Expression profiles of organ-specific miRNAs

**miRNAs**	**Signal intensity across all organs**					**Highly-expressed organ(s)/tissue(s)**	**Human expression report**
	**Min.**	**25%**	**Med.**	**75%**	**Max.**		
rno-miR-7a	17.69	40.39	73.38	254.21	21,016	Pituitary gland, cerebrum_thalamus, adrenal gland, lymph node_cervical, lymph node_mesenteric	Bottoni, A. *et al.* (^22^)
rno-miR-34b	0.09	0.30	9.52	25.97	12,120	Testis, trachea, lung, epididymis	Liang, Y. (^23^)
rno-miR-122	0.04	0.10	0.17	0.27	237,949	Liver	Starkey Lewis, P. J. *et al.* (^16^)
rno-miR-124	0.04	0.14	0.24	0.66	21,435	Cerebellum, eyeball, cerebrum_cerebral cortex, cerebrum_hippocampus, medulla oblongata	Smirnova, L. *et al.* (^24^)
rno-miR-128	0.66	61.76	110.78	493.04	10,167	Cerebrum_cerebral cortex, cerebellum, cerebrum_hippocampus, thymus, medulla oblongata	Smirnova, L. *et al.* (^24^)
rno-miR-142-3p	0.69	138.15	336.86	746.27	53,949	Lymph node_cervical, thymus, lymph node_mesenteric, spleen, bone marrow	No report
rno-miR-144	0.04	0.12	0.20	0.64	3,291	Bone marrow, spleen	No report
rno-miR-150	0.66	105.59	216.92	582.12	14,087	Lymph node_mesenteric, lymph node_cervical, spleen, thymus, brown adipose tissue	No report
rno-miR-181b	0.33	49.48	93.90	144.48	2,640	Thymus, optic nerve, bone marrow, medulla oblongata, spinal cord_cervical	No report
rno-miR-184	0.04	0.11	0.17	0.28	14,536	Eyeball	Tian, L. *et al.* (^25^)
rno-miR-192	0.22	16.65	31.09	53.94	38,628	Intenstines (duodenum, jejunum, ileum, cecum, colon, rectum), stomach_glandular, liver, kidney	Jenkins, R. H. *et al.* (^26^)
rno-miR-206	0.04	0.12	0.23	8.49	28,471	Skeletal muscles (musculus soleus, femoris muscle, gastrocnemial muscle), interseptum, esophagus, tongue	Nielsen, S. *et al.* (^27^)
rno-miR-208_v15.0	0.04	0.10	0.15	0.27	939	Heart_interventricular septum, heart_atrium	van Rooij, E. *et al.* (^28^)
rno-miR-215	0.04	0.10	0.19	0.32	50,159	Duodenum, jejunum, ileum, rectum, colon	Sharbati, S. *et al.* (^29^)
rno-miR-216a	0.04	0.11	0.16	0.26	2,713	Pancreas	Szafranska, A. E. *et al.* (^30^)
rno-miR-219-2-3p	0.04	0.13	0.20	0.34	1,915	Optic nerve, spinal cord_cervical, spinal cord_pectoral, medulla oblongata, spinal cord_pars lumbalis	No report
rno-miR-499	0.11	8.57	17.70	43.21	2,233	Heart_interventricular septum, heart_atrium, interseptum, musculus soleus	Sluijter, J. P. *et al.* (^31^)
rno-miR-542-3p	0.05	0.32	9.15	29.89	439	Adrenal gland	No report
rno-miR-551b	0.04	0.15	0.24	0.72	1,607	Pituitary gland, cerebrum, medulla oblongata, spinal cord	No report
rno-miR-652	120.14	223.45	322.41	550.64	8,315	Harderian gland, bone marrow, adrenal gland, rectum, lung	No report
